# Genome-wide promoter analysis, homology modeling and protein interaction network of Dehydration Responsive Element Binding (DREB) gene family in *Solanum tuberosum*

**DOI:** 10.1371/journal.pone.0261215

**Published:** 2021-12-16

**Authors:** Qurat-ul ain-Ali, Nida Mushtaq, Rabia Amir, Alvina Gul, Muhammad Tahir, Faiza Munir

**Affiliations:** Department of Plant Biotechnology, Atta-ur-Rahman School of Applied Biosciences, National University of Sciences and Technology, Islamabad, Pakistan; Government College University Faisalabad, PAKISTAN

## Abstract

Dehydration Responsive Element Binding (DREB) regulates the expression of numerous stress-responsive genes, and hence plays a pivotal role in abiotic stress responses and tolerance in plants. The study aimed to develop a complete overview of the cis-acting regulatory elements (CAREs) present in *S*. *tuberosum* DREB gene promoters. A total of one hundred and four (104) cis-regulatory elements (CREs) were identified from 2.5kbp upstream of the start codon (ATG). The in-silico promoter analysis revealed variable sets of cis-elements and functional diversity with the predominance of light-responsive (30%), development-related (20%), abiotic stress-responsive (14%), and hormone-responsive (12%) elements in StDREBs. Among them, two light-responsive elements (Box-4 and G-box) were predicted in 64 and 61 StDREB genes, respectively. Two development-related motifs (AAGAA-motif and as-1) were abundant in StDREB gene promoters. Most of the DREB genes contained one or more Myeloblastosis (MYB) and Myelocytometosis (MYC) elements associated with abiotic stress responses. Hormone-responsive element i.e. ABRE was found in 59 out of 66 StDREB genes, which implied their role in dehydration and salinity stress. Moreover, six proteins were chosen corresponding to A1-A6 StDREB subgroups for secondary structure analysis and three-dimensional protein modeling followed by model validation through PROCHECK server by Ramachandran Plot. The predicted models demonstrated >90% of the residues in the favorable region, which further ensured their reliability. The present study also anticipated pocket binding sites and disordered regions (DRs) to gain insights into the structural flexibility and functional annotation of StDREB proteins. The protein association network determined the interaction of six selected StDREB proteins with potato proteins encoded by other gene families such as MYB and NAC, suggesting their similar functional roles in biological and molecular pathways. Overall, our results provide fundamental information for future functional analysis to understand the precise molecular mechanisms of the DREB gene family in *S*. *tuberosum*.

## Introduction

Potato *(Solanum tuberosum)* is an important agricultural crop worldwide only after wheat and rice [1]. According to FAO estimates, approximately 370 million metric tons of potatoes were produced worldwide in 2019, a substantial rise from the 333.6 million tons produced in 2010 [2]. However, potato is extremely sensitive to various abiotic stresses especially drought [3]. Potato susceptibility to drought has been predominantly linked to its shallow root system [4], with root length of cultivar being correlated with production under drought conditions [5] and canopy features [6], with -stem-type canopy- varieties showing more tolerance to drought than leaf-types [7]. These features can cause a dramatic decrease in yields under water scarcity, with a previous study reporting an 87% reduction in tuber yields in the Desiree cultivar [7]. Globally it is estimated that drought will decrease potato yield by 32% between 2040 and 2069[8].

Plant transcription factors (TFs) are a class of proteins that specifically binds to cis-acting regulatory elements (CREs) present in the promoter regions of the targeted genes and regulate gene expression by extracellular and intercellular signaling [9]. Moreover, TFs are considered as one of the most potential candidates for genetic engineering in plants [10].

Dehydration Responsive Element Binding (DREB) transcription factor family belongs to the APETALA2/ ethylene responsive element binding protein (AP2/EREBP) superfamily. DREB genes consist of a highly conserved DNA-binding AP2 domain [11]. Furthermore, the DREB gene family members are defined by the presence of Valine (V) at position 14^th^ and glutamic acid (E) at position 19^th^ within the conserved AP2 domain [12]. Based on the genetic domain, DREB genes can be classified into six subgroups i.e. A1 to A6. Members of the DREB gene family are known for their regulatory roles in various abiotic stresses such as drought [13], salinity [14], heat [15], and cold [16]. DREB genes enhance a plant’s abiotic stress tolerance by interacting with core dehydration responsive element (DRE) sequence (5’-A/GCCGAC-3’) cis-element located in their promoter region. The specificity and strength of promoter regions are defined by the architecture of their CREs [17]. Thus, gene promoters are central for the regulation of gene expression in response to abiotic stresses [18]. Recent advances in experimental techniques such as RNA sequencing, microarray, and RNA interference have enabled the discovery and exploration of target gene promoter regions; however these techniques are expensive and technically demanding [19, 20]. Therefore, computational approaches are being utilized to scan the promoter regions for various cis-elements involved in gene regulation [21]. Different web-based tools have been developed to analyze cis-acting elements within the promoter regions for example PlantCARE [22], Plant Pan [23], Plant Prom [24], AGRIS [25], and PLACE [26]. Various types of cis-acting elements associated with different gene families have been reported in plant species such as auxin responsive elements (AuxRE) in *A*. *thaliana* and rice [27]. Furthermore, hormone, light, and stress-responsive cis-regulatory elements have been identified for basic helix-loop-helix (bHLH) [28], basic leucine zipper (bZIP), and glycogen synthase kinase (GSK) gene families in potato [29, 30].

With the completion of the whole genome sequencing (WGS), DREB gene family members have been identified and characterized in many plant species including soybean [31], mulberry [32], malus [33], and common bean [34]. DREB TFs have been reported to play key roles in drought, salinity, heat, and cold in *Solanum tuberosum* [35–37]. Considering the significance of DREB genes in plant abiotic stress tolerance, we conducted a genome-wide screening of the potato genome in our recently published research study. A total of sixty-six (66) StDREB genes were identified and classified into six distinct subgroups (A1-A6) with reference to the classification of *Arabidopsis* DREB genes. Detailed motif patterns and exon/intron organization was analyzed, which was found consistent with phylogenetic classification. All the sixty-six (66) DREB genes were mapped across 12 potato chromosomes. Gene duplication suggested that DREB genes had undergone both tandem and segmental duplications during evolution. According to subcellular localization, the majority of the StDREB genes were found in the nucleus. Moreover, we performed functional annotation and concluded the DNA-binding ability of each *S*. *tuberosum* DREB gene [38]. To the best of our knowledge, no study has been reported on cis-elements of DREB genes in *S*. *tuberosum*. In the present study, genome-wide cis-elements were predicted in the promoter regions of StDREB genes. We investigated 104 CREs of the DREB gene family in the potato genome along with their potential functions through in-silico approaches. We performed homology modeling of six StDREB proteins from A1-A6 subgroups and validated the predicted models to gain an in-depth knowledge of structural variations and functional stability. Furthermore, we analyzed interaction network of potato DREB proteins with potential regulatory proteins to predict the possible regulation relationship between DREBs and various protein families. Collectively, our findings provide useful insights for further functional investigations of DREB genes and multi-stress tolerance engineering in *S*. *tuberosum*.

## Materials and methods

### Cis-regulatory element analysis of StDREB gene promoters

In our recently published study, we identified a total of sixty-six (66) DREB genes in the *S*. *tuberosum* genome [38]. To investigate cis-regulatory elements in promoter regions of 66 StDREB genes, promoter sequences 2.5 kbp upstream of the translational start site (ATG) were retrieved from Phytozome v12.1 database (https://phytozome.jgi.doe.gov/pz/portal.html) for each StDREB gene [39]. The promoter sequences were then submitted to the PlantCARE database (http://bioinformatics.psb.ugent.be/webtools/plantcare/html/) [40] and validated in NEW PLACE (https://www.dna.affrc.go.jp/PLACE/?action=newplace) to predict diverse cis-regulatory motifs of the DREB gene family in *S*. *tuberosum*. Furthermore, based on the initiation and termination positions of the respective motifs, the CREs and their associated DREB genes were illustrated using ToolKit Biologists Tools (TB tools) software (https://github.com/CJ-Chen/TBtools).

### Three-dimensional (3D) molecular modeling and structural validation of StDREB proteins

For homology modeling, the full-length amino acid sequences of six representative StDREB proteins from A1-A6 subgroups were retrieved using the UNIPROT database (https://www.uniprot.org/) [41]. To conduct the secondary structure analysis of StDREB proteins, the SOPMA server (https://npsa-prabi.ibcp.fr/NPSA/npsa_sopma.html) was employed [42]. The three-dimensional structures of StDREB proteins were predicted by Protein Homology/ analogy Recognition Engine V 2.0 (Phyre2) server (http://www.sbg.bio.ic.ac.uk/~phyre2/html/page.cgi?id=index) that uses advanced homology detection methods to generate 3D models [43]. The predicted PDB models were then refined by the Galaxy Refine server (http://galaxy.seoklab.org/cgi-bin/submit.cgi?type=REFINE) [44]. Structural validation of StDREB proteins was performed using PROCHECK server with Ramachandran plot analysis (https://servicesn.mbi.ucla.edu/PROCHECK/).

### Prediction of the active catalytic site and disordered regions of StDREB proteins

The multiscale pocket structures on StDREB protein surfaces were determined using CASTp 3.0 (Computed Atlas of Surface Topography of proteins) (http://sts.bioe.uic.edu/castp/index.html?2pk9) web-server [45]. The disordered regions were predicted using PrDOS (Protein DisOrder prediction System) server (http://prdos.hgc.jp/cgi-bin/top.cgi) respectively [46].

### Interaction network analysis of StDREBs

The STRING tool (Search Tool for the Retrieval of Interacting Genes) (https://string-db.org/) [47] was employed to construct the protein association network between StDREBs and their interacting protein families, with a confidence parameter set to a threshold of 0.40, a maximum number of interactors in the first shell limited to 10, and none in the second shell respectively. The amino acid sequences were used as query subjects to analyze the network model of putative interacting partners. The proteins that belonged to the same sub-network were interconnected to each other.

## Results and discussion

In the present study, an effort has been made to analyze various cis-regulatory elements (CREs) found in *Solanum tuberosum* DREB gene promoters, the 3D protein structures of StDREB proteins, structural validation, as well as their association with other transcription factor families in a regulatory network.

### Identification of cis-regulatory elements in the promoter regions of StDREB genes

**Fig 1:**
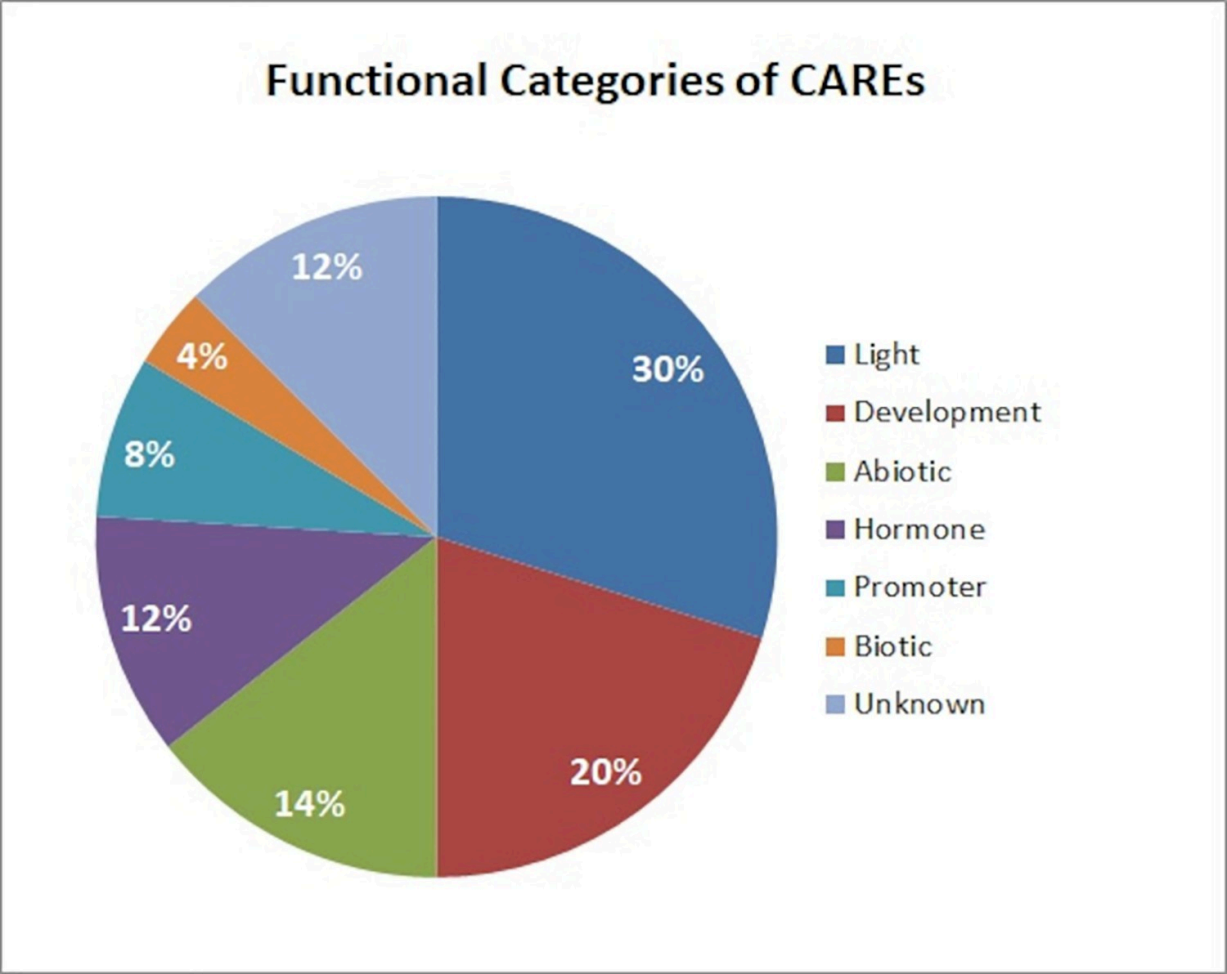
Functional classification of predicted cis-elements in StDREB promoter regions. Different colors indicated the frequency of cis-elements namely light responsive elements (30%); development-related elements (20%); abiotic stress-responsive elements (14%); hormone-responsive elements (12%); promoter-related elements (8%); biotic stress-responsive elements (4%); and elements with unknown function (12%).

**Fig 2:**
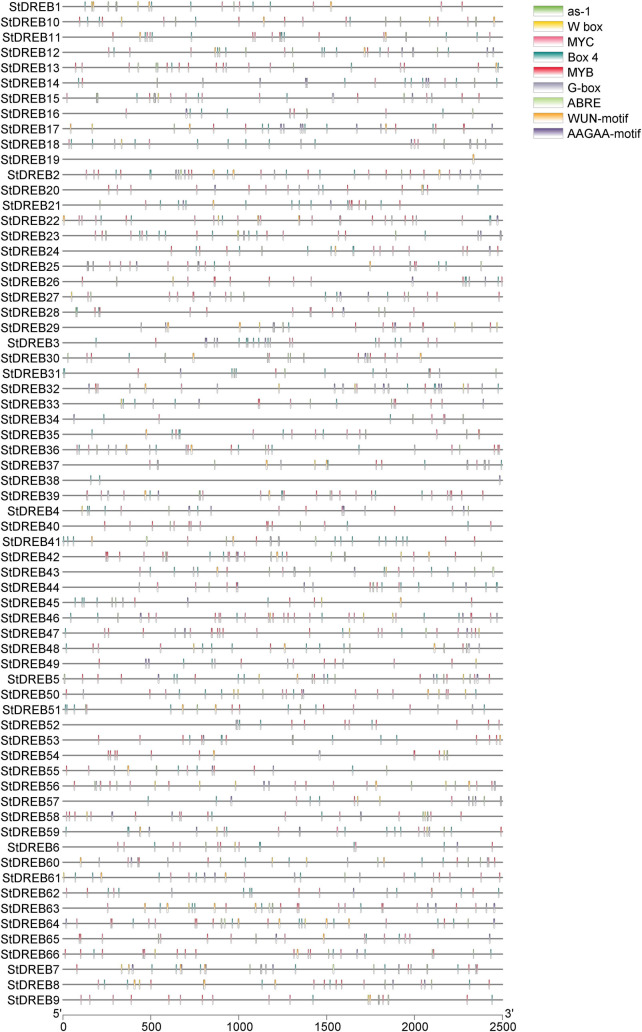
Distribution of frequently occurring CREs in sixty-six (66) StDREBs. They were visualized using Simple BioSequence Viewer in TB tools software. Different colors demonstrated nine types of motifs. Bars at the right represented names of nine motifs that respond to light stress (Box 4 and G box), plant development (as-1, AAGAA-motif), hormone signaling (ABRE), abiotic stress (MYB, MYC), and biotic stress (W box, WUN-motif). The scale at the bottom indicated the length of the promoter.

DREB gene family is defined by the presence of an AP2 domain [48]. All of the retrieved DREB genes were verified for the presence of one conserved AP2 domain followed by amino acid conservation analysis i.e. V14 and E19 [38]. The presence of valine (V) at position 14^th^ and glutamic acid (E) at position 19^th^ play central roles in consensus recognition and binding to the DRE cis-element, however, E might show some flexibility [49]. A total of 104 cis-elements were identified from the 2.5 kbp upstream region of the DREB gene family by the PlantCARE program and their respective functions were validated in the NEW PLACE database (S1 Table). Predicted cis-regulatory elements were grouped into seven broad categories (Fig 1); based on their function and response to stimuli namely, light responsive, plant growth and development, abiotic stress responsive, hormonal regulation, promoter related, biotic stress responsive, and cis-elements with unknown functions. Statistical analysis demonstrated that light responsive elements comprised 30% of all CREs, followed by development related elements (20%), abiotic stress responsive elements (14%), and hormone responsive elements (12%), suggesting the involvement of DREB in photoperiod responses, plant development, hormonal regulation and in providing tolerance to plants against abiotic stresses. This result is consistent with the previously performed in vivo studies on DREB genes [15]. Furthermore, biotic stress responsive elements represented the lowest proportion (4%) of the CREs. Promoter related cis-elements accounted for 8% of the total while 12% of members had unknown functions. The length of cis-acting elements varied from 4–13 bp in *S*. *tuberosum*. The majority of the cis-elements were 4 and 6 bp in length. The highest number of motifs was observed for the promoter of StDREB37 while the lowest number of motifs was found in the promoter of StDREB38 (S2 Table). Fig 2 represents the distribution of frequently occurring cis-acting motifs with their corresponding StDREB genes. Box 4 and G-box are light responsive cis elements present in DREB promoters, whereas activating sequence-1 (as-1) and AAGAA-motif are developmental-related elements. The as-1 is present in a large number of promoters induced by stress, including the pathogenesis-related protein 1 promoter [50, 51]. For instance, as-1 elements have been found in pathogenesis related promoters of *Arabidopsis* and tobacco [52]. The as-1 elements are also reported to be involved in root specific expression [53]. MYC and MYB are dehydration responsive elements residing in DREB gene promoters establishing their role against drought. ABRE is an abscisic acid responsive element, suggesting the role of DREB genes in abiotic stress signaling e.g. drought. W box is elicitor responsive cis-element present in the maximum number of 4 in StDREB32, StDREB50, and StDREB56 gene respectively. W boxes interact with WRKY transcription factors to induce stress responsive genes against biotic and abiotic stresses [54]. The presence of wound responsive elements (WUN-motif) in StDREB genes depicts their role against biotic stresses. Overall, the presence of different cis-elements indicates the functional diversity of StDREB genes that ultimately reveal their biological importance in helping plants to cope with environmental stresses.

#### Promoter-related elements

**Fig 3:**
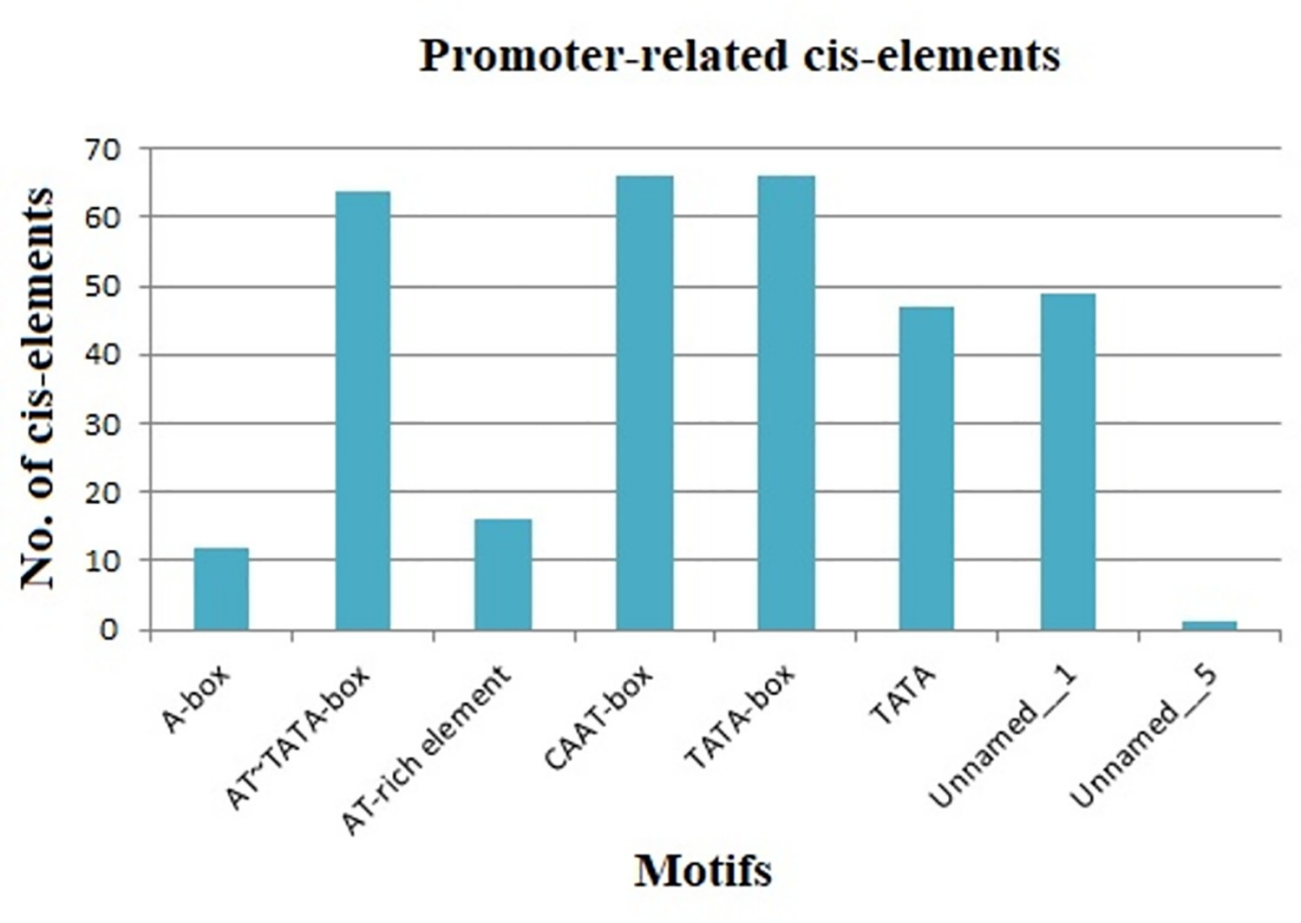
Graphical representation of promoter-related elements in StDREB genes identified using PlantCARE and NEW PLACE database. X-axis represented names of various motifs, whereas Y-axis represented the number of cis-elements in 66 StDREB genes.

Based on the results, a total of 8% of the 104 cis-elements belonged to promoter-related elements. We observed the presence of two core promoter elements in all 66 StDREB genes, namely CAAT-box and TATA-box, both of which act as binding sites for a transcription factor. TATA-box is essential for recruiting the basal machinery and determining the transcriptional start site (TSS) [55, 56], while CAAT-box is the major determinant of promoter efficiency due to its sensitivity to mutations [57]. Table 1 shows the frequency of presence of two core promoter elements in each StDREB promoter region. Besides the two core promoter elements, A-box, AT-rich elements, AT~TATA-box, TATA, unnamed_1, and unnamed_5 were also predicted as promoter binding sites. In tobacco, NtAIDP1 binds to the AT-rich region in the promoter of JA-responsive genes to activate transcription [58]. The A-box for α-amylase promoters is previously identified as stress-relevant in the promoters of zinc finger proteins Zat7 and Zat12, as well as WRKY 25 transcription factor [59]. Our results indicated the existence of AT~TATA-box in 64 StDREB genes, unnamed_1 in 49 StDREB genes, and TATA in 47 StDREB genes whereas, AT-rich elements were found in 16 StDREB genes, A-box in 12 StDREB genes, and unnamed_5 in one StDREB gene only (Fig 3).

**Table 1:** Frequency of occurrence of CAAT-box and TATA-box in sixty-six (66) StDREB genes.

Gene name	CAAT Box	TATA Box	Gene name	CAAT Box	TATA Box
**StDREB1**	43	76	**StDREB34**	25	133
**StDREB2**	44	95	**StDREB35**	38	73
**StDREB3**	58	67	**StDREB36**	47	90
**StDREB4**	51	90	**StDREB37**	50	114
**StDREB5**	45	100	**StDREB38**	11	6
**StDREB6**	55	100	**StDREB39**	67	77
**StDREB7**	33	91	**StDREB40**	53	77
**StDREB8**	57	74	**StDREB41**	38	89
**StDREB9**	54	37	**StDREB42**	45	86
**StDREB10**	55	69	**StDREB43**	40	86
**StDREB11**	42	47	**StDREB44**	47	46
**StDREB12**	46	96	**StDREB45**	48	58
**StDREB13**	39	107	**StDREB46**	61	91
**StDREB14**	50	108	**StDREB47**	51	58
**StDREB15**	40	68	**StDREB48**	46	73
**StDREB16**	42	103	**StDREB49**	30	57
**StDREB17**	46	50	**StDREB50**	37	80
**StDREB18**	54	67	**StDREB51**	49	114
**StDREB19**	12	27	**StDREB52**	67	75
**StDREB20**	51	65	**StDREB53**	49	90
**StDREB21**	36	98	**StDREB54**	45	22
**StDREB22**	59	85	**StDREB55**	53	59
**StDREB23**	40	113	**StDREB56**	48	58
**StDREB24**	62	113	**StDREB57**	53	90
**StDREB25**	60	43	**StDREB58**	44	80
**StDREB26**	40	24	**StDREB59**	49	71
**StDREB27**	66	57	**StDREB60**	57	102
**StDREB28**	52	94	**StDREB61**	49	72
**StDREB29**	52	42	**StDREB62**	47	94
**StDREB30**	49	82	**StDREB63**	55	57
**StDREB31**	67	82	**StDREB64**	65	63
**StDREB32**	50	88	**StDREB65**	39	58
**StDREB33**	46	83	**StDREB66**	58	75

#### Light responsive cis-elements

**Fig 4:**
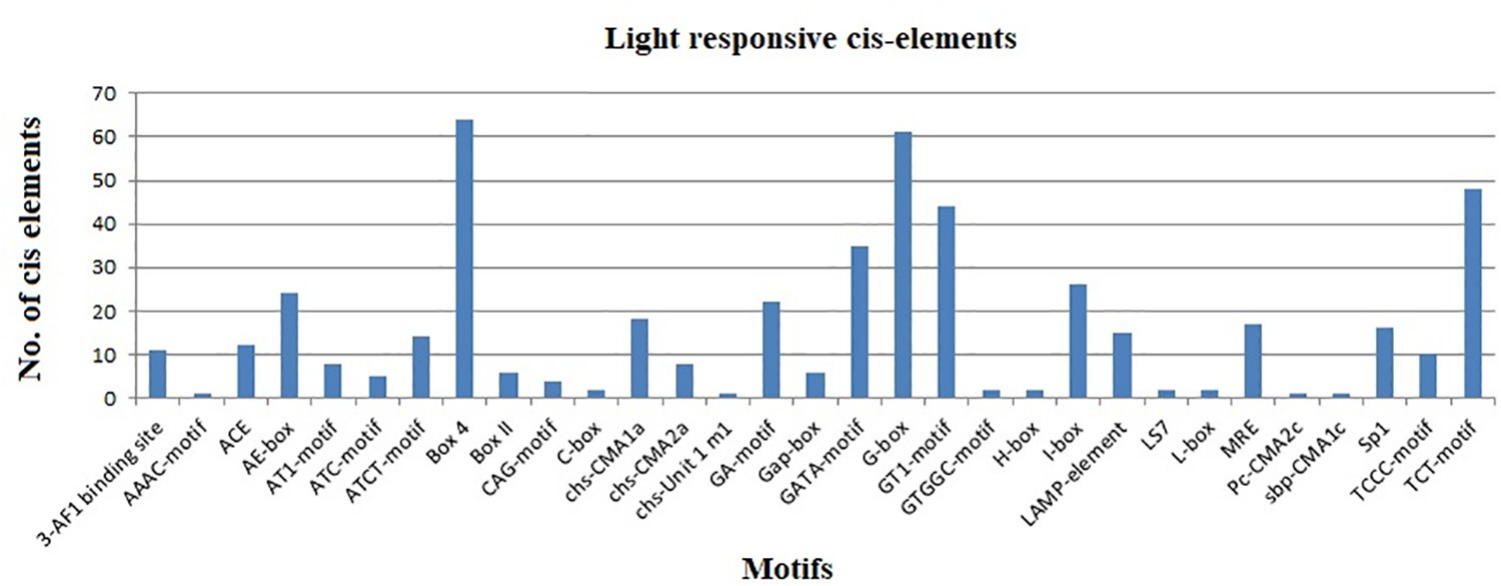
Light responsive motifs identified in *S*. *tuberosum* DREB gene promoters. Promoter sequences were submitted in the PlantCARE database to predict motifs and validated through the NEW PLACE database. Results showed 21 types of motifs involved in light responsiveness with Box 4 and G Box showing maximum frequency.

The assessment of *S*. *tuberosum* DREB gene promoters revealed thirty-one light-responsive elements (LREs), such as Box-4, G-box, C-box, GATA-motif, GA-motif, Gap-box, TCT-motif, ATCT-motif, 3-AF1 binding site, AAAC-motif, ACE, Box II, CAG-motif, chs-CMA1a, chs-CMA2a, chs-Unit 1 m1, AE-box, AT1-motif, ATC-motif, TCCC-motif, LAMP-element, LS7, MRE, Pc-CMA2c, sbp-CMA1c, GT1-motif, GTGGC-motif, H-box, I-box, L-box, and Sp1 (Fig 4). Almost all StDREB genes contained at least one light-responsive element in their promoter regions. Among currently identified LREs many elements including G-Box, ACE, Box-4, Sp1, TCT-motif, GATA motif have been also previously identified in the promoters of the drought, salinity, cold, and heat responsive genes [60]. These elements perform a crucial role in regulating transcriptional activity [61]. Light responsive elements such as Box-4, G-Box are well known to be present in the regulatory areas of those genes that regulate light-controlled transcriptional activities [62]. Two motifs i.e. Box-4 and G-box were prevalent cis-elements predicted in the promoter regions of 64 and 61 genes of the total 66 StDREBs, respectively. Box 4 is a part of the conserved DNA module involved in light-responsiveness. It is worth mentioning that the Box-4 element is enriched in the soybean WRKY genes, suggesting that the Box-4 element is crucial for light-regulated transcriptional activity [63]. G-box element is a conserved region located upstream of light-regulated genes that function as a molecular switch activated by calcium dependent phosphorylation/dephosphorylation in response to light signals. The G-box provides binding sites for specific bZIP proteins and this cis-element is regulated to environmental stresses such as UV light, ABA, red light, and injuries [64]. It has been reported that G-box is common in *Arabidopsis* and grapevine and is involved in photosynthesis, hormone signaling (ethylene and ABA), and stress responses [65]. According to a study on rice, G-box is the cognate cis-element for bHLH, bZIP, and NAC TFs, implying a role in the regulation of transcriptional activity [66]. Further, G-box has been identified as a MYC recognition site along with MYB, playing a role in the upregulation of the RD22 drought-induced gene [67]. Furthermore, GATA-motif is considered essential for high level light-regulated and tissue specific expression [68]. The presence of TCT motif indicates the role of DREBs in photoperiod responses. The previous study has proved the role of TCT motif as a positive regulator of photoperiod by causing early flowering and short growth period in *Arabidopsis* [69]. The GT-1 motif specifically binds to the light induced genes and is known to enhance light responses [70, 71]. According to a study on rice, GT-1 serves as a crucial cis-acting element that regulates Os2H16 expression in response to osmotic stress and pathogen attack [72]. A previous study also confirmed that 3-AF1 binding site and I-box are related to light responses [73]. Interestingly, the C-box cis-element was only present in StDREB63 and StDREB64 gene promoter regions. Each StDREB gene exhibited at least one type of light-responsive cis-element in the promoter region which strongly indicates their association with photoperiod control of flowering.

#### Role of DREB cis-elements in developmental processes

**Fig 5:**
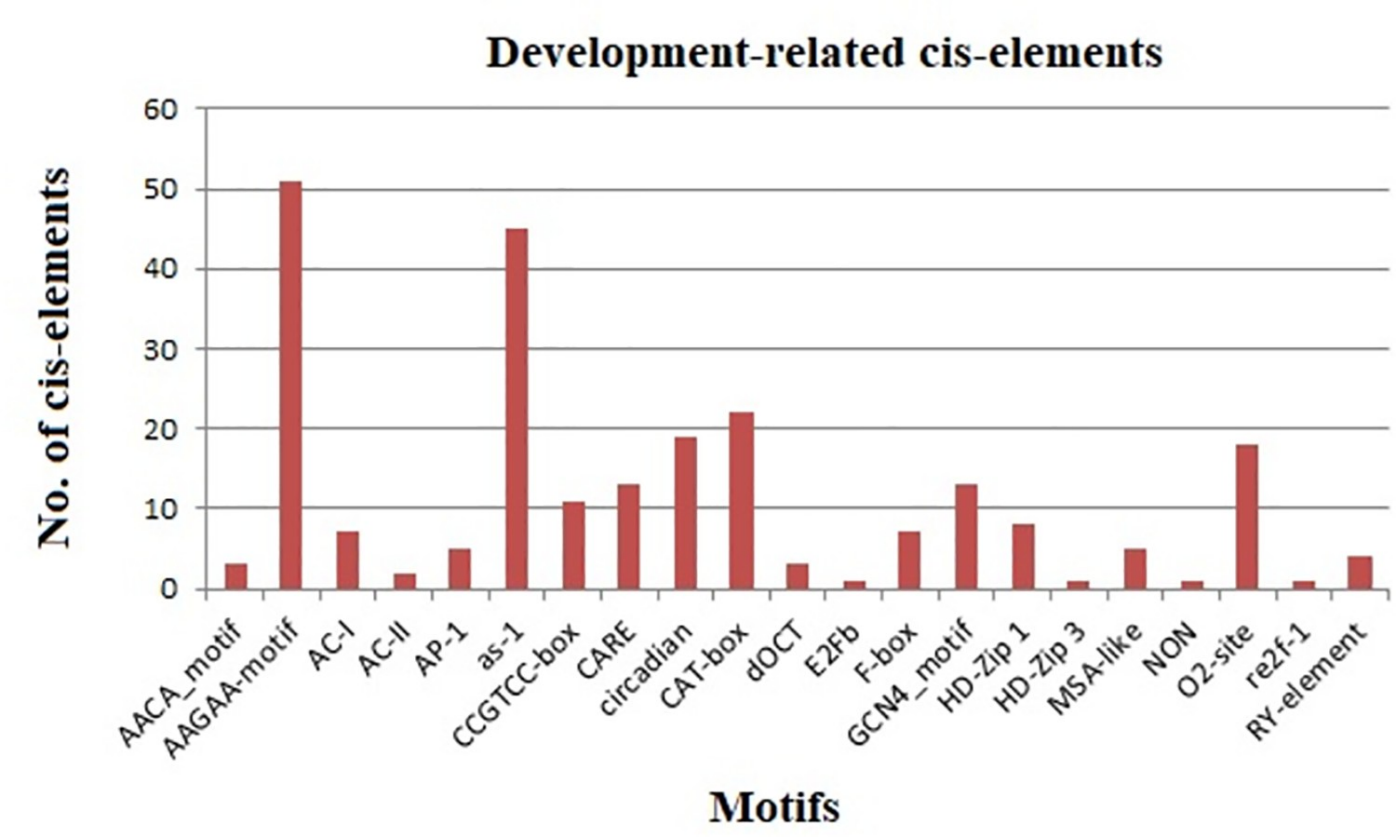
Histogram based representation of developmental-related elements in StDREBs. Results revealed 21 types of motifs with AAGAA-motif and as-1 showing maximum percentage in StDREB gene promoters.

The results showed that the promoters of StDREBs contained twenty-one CREs including AACA_motif, AAGAA-motif, AC-I, AC-II, AP-1, as-1, CCGTCC-box, CARE, circadian, CAT-box, dOCT, E2Fb, F-box, GCN4_motif, HD-Zip 1, HD-Zip 3, MSA-like, NON, O2-site, re2f-1, and RY-element. Motifs involved in growth and developmental processes were found to be the second largest in number after light-responsiveness. Fig 5 represents development related CREs present in 66 StDREB genes. The regulatory role of each anticipated motif was determined. AAGAA-motif and as-1 motif were in maximum abundance in StDREB gene promoter regions. The AAGAA-motif was found to be associated with secondary xylem development while the motif as-1 was found to be linked with transcriptional activity of several genes mediated by auxin and salicylic acid. Our results revealed the presence of AACA_motif and GCN4_motifs having a frequency of 4.5% and 24.2% respectively, which were found responsible for endosperm expression. Other elements consisted of O2-site, circadian, RY-element, MSA-like, CAT-box, and HD-ZIP 1 which were found to be involved in zein metabolism, circadian control, seed-specific regulation, cell cycle activity, meristem expression, and differentiation of palisade mesophyll cells respectively. Both CARE and CAT-box were considered important for meristem-specific expression. CAT-box signals the binding site for RNA transcription factors, acting as a key regulator of gene expression [74]. The motifs AC-I and AC-II had roles in xylem-specific expression. Moreover, E2Fb was only identified in one gene i.e. StDREB7 promoter while HD-ZIP 3 motif was only recognized in the promoter region of StDREB11 (S2 Table). E2Fb cis-element is found to be responsible for cell regulation [75] whereas, the HD-ZIP3 motif is linked with cambium and vascular development [76]. Our results were found consistent with the previous studies conducted in the *Saccharum spontaneum* DREB gene family with the exception of only five cis-acting elements (AC-II, AACA_motif, CARE, F-box, and HD-Zip 3) [77].

#### Stress responsive cis-elements

**Fig 6:**
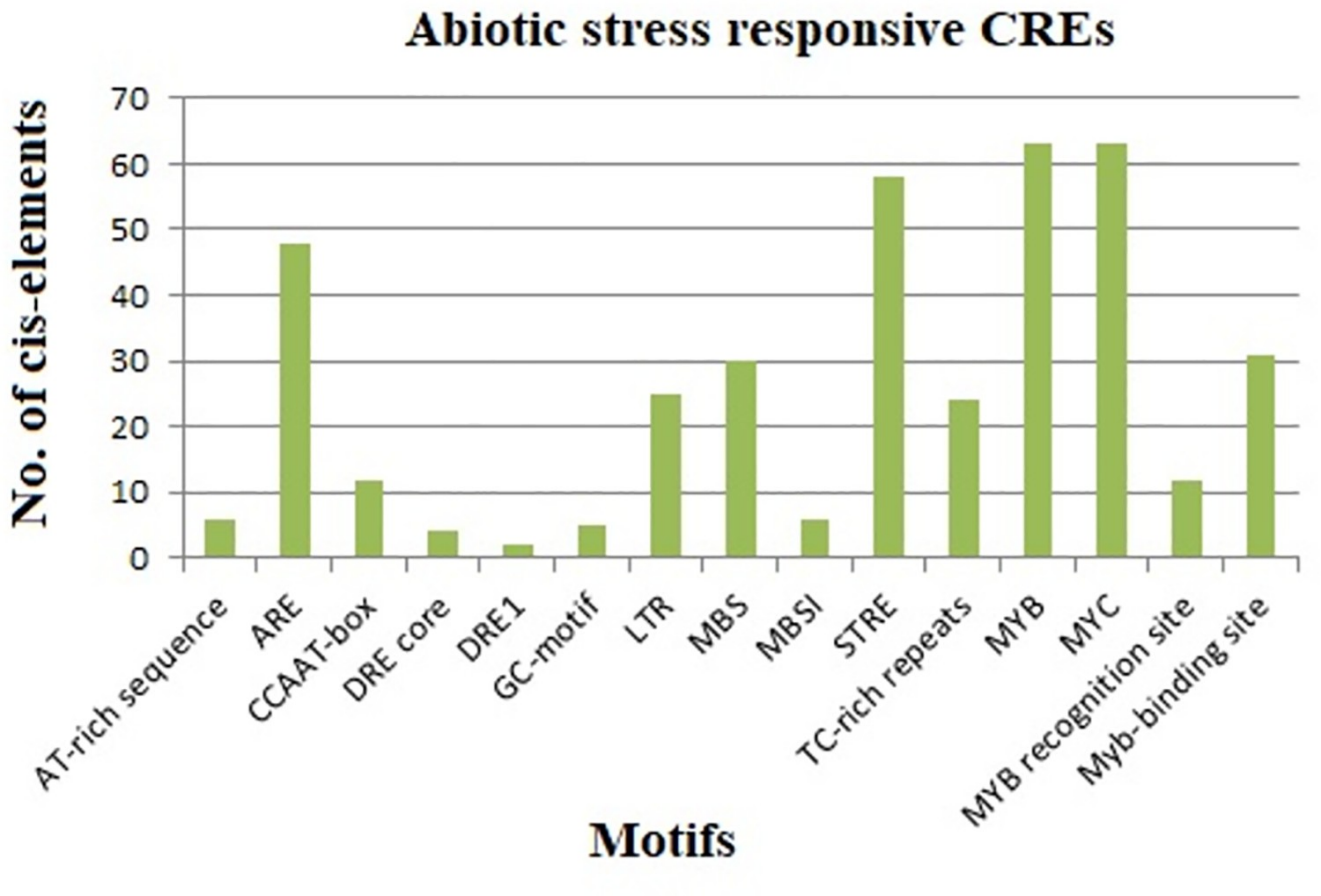
Fifteen types of abiotic stress responsive elements in StDREB gene promoters. Four cis-elements including MYC, MYB, STRE, and ARE showed the highest percentage in DREB gene promoter regions.

Abiotic stress serves as a major challenge in potato production. The abiotic stress responsive category consisted of 15 types of motifs, such as AT-rich sequence, ARE, CCAAT-box, DRE core, DRE1, GC-motif, LTR, MBS, MBSI, STRE, TC-rich repeats, MYB, MYC, MYB recognition site, and Myb-binding site (Fig 6). Among the stress responsive elements identified in StDREB genes ARE, STRE, MYC and MYB were found in the highest frequency of 71%, 87%, 95%, and 96% respectively (S2 Table). Functional analysis demonstrated the correlation of CCAAT-box with heat shock elements (HSEs) to increase heat shock promoter activity [78]. AT-rich sequence is linked with elicitor-mediated activation during stress responses whereas GC-motif is an enhancer like element involved in anoxic specific inducibility [79]. The ARE motif is an essential regulatory element for anaerobic induction. AREs (Anaerobic responsive elements) structure analysis shows that they are bipartite elements comprising of GT and GC motifs. GT motif show resemblance with MYB2 transcription binding site, which is low oxygen and dehydration induced element [80]. The results revealed that sixty-three (63) out of sixty-six (66) DREB gene members consisted of one or more MYB (drought-responsive) and MYC (dehydration-responsive) elements in their promoters indicating that StDREB expression is associated with abiotic stress. MYB and MYC are considered as important regulators of the plant stress response. MYB transcription factors play a central role in plant development, secondary metabolism, hormone signal transduction, abiotic stress tolerance, and disease resistance [81]. However, MYB genes have a predominant role in drought stress tolerance. MYB transcription factors require MBS for the gene expression of drought inducible genes [82]. On the other hand, MYC transcription factors are important regulators of the jasmonic acid (JA) signaling pathway. JA participates in the regulation of diverse processes encompassing plant growth and development, secondary metabolite biosynthesis, wounding response, and biotic and abiotic stresses [83]. Numerous CREs such as STRE (stress-response element), DRE (dehydration responsive element), LTR (low temperature responsive), and MBS (Myb binding site) have been identified as drought inducible cis-motifs implicated in the drought responsive gene regulation in plants [84]. According to a study on sweet potatoes, STRE is involved in heat stress responses, which typically regulate genes in response to heat stress [85]. Thus, our findings showed the ability of the StDREB genes to bind promoter sequences and respond to various abiotic stresses.

**Fig 7:**
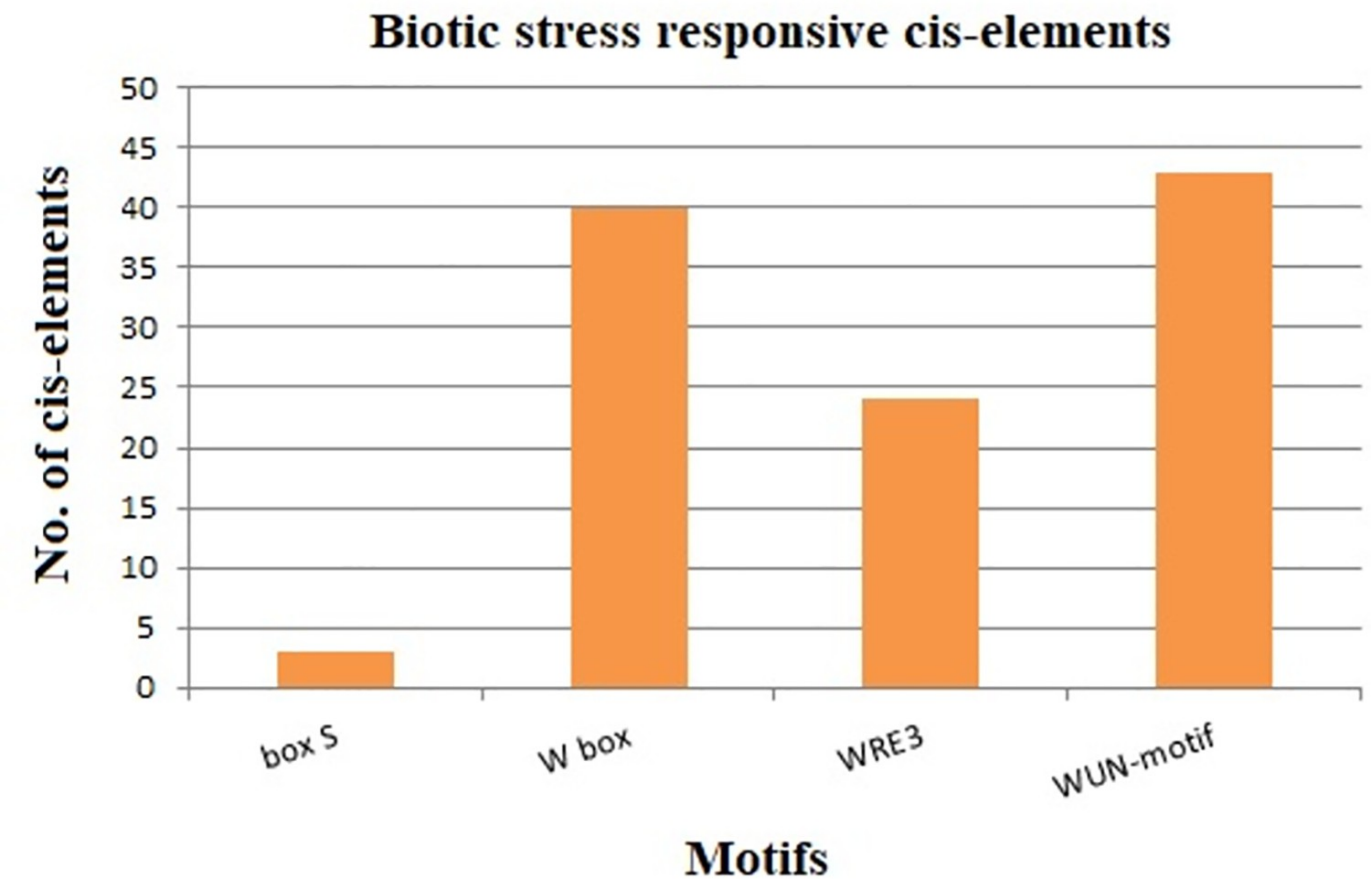
Biotic stress responsive elements identified in sixty-six (66) StDREB promoter regions. Results demonstrated four types of motifs involved in biotic stress responses with WUN-motif and W-box found in the highest percentage.

Moreover, the existence of box S, WRE3, W-box, and WUN-motif in promoters suggested that DREB genes might play a vital role in biotic stress responses (Fig 7). WUN-motif (wound-responsive element) was found in 43 StDREB gene promoters while W-box was present in 40 StDREB genes of the total 66 StDREBs (S2 Table). W-box is a fungal elicitor responsive cis-element. W-boxes have been discovered to interact with WRKY transcription factors [66]. According to previous studies, *Saccharum spontaneum* DREB genes are also comprised of these biotic stress-responsive cis-elements whereas Pineapple consisted of W-box only [86].

#### Role of cis-elements in phytohormone regulation

**Fig 8:**
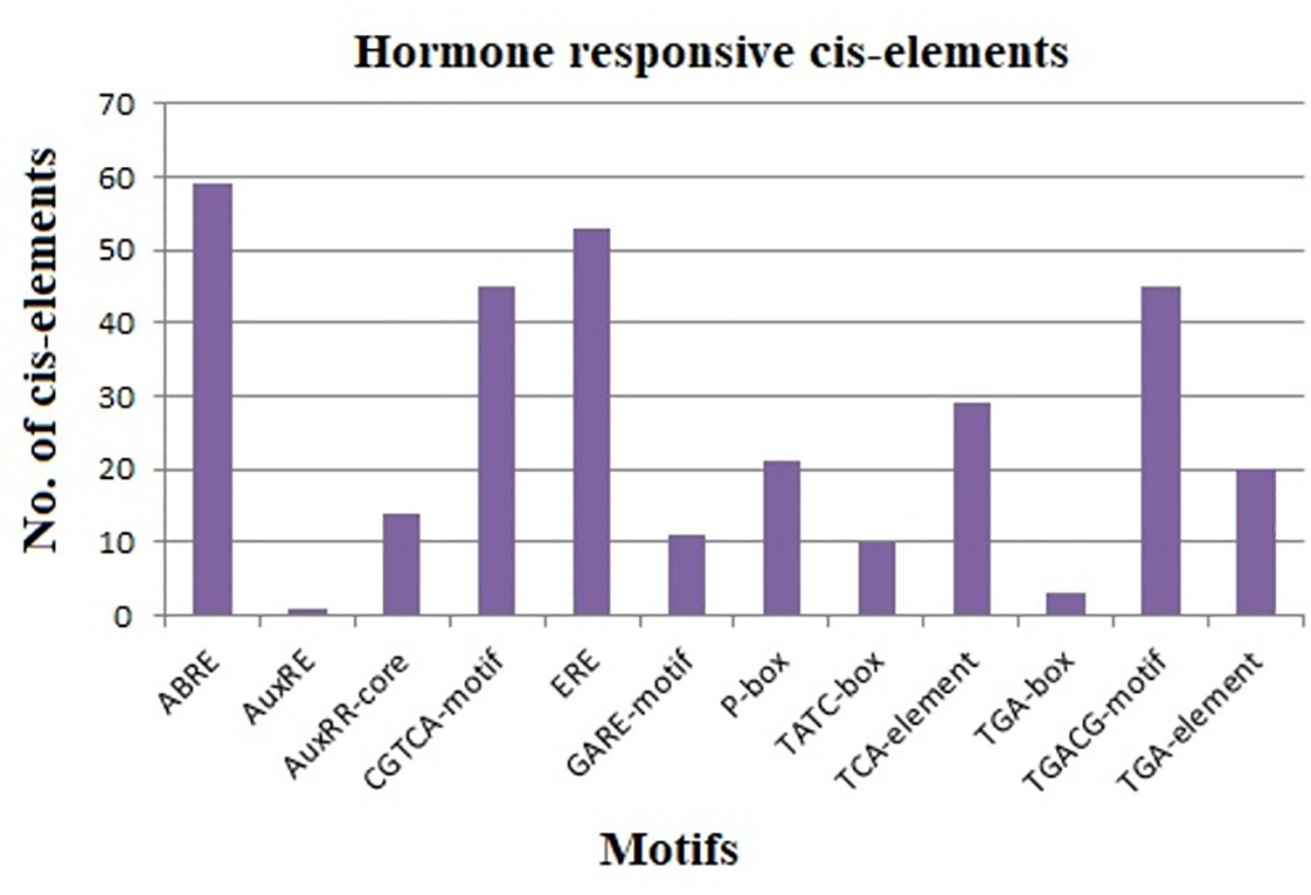
Graphical illustration of hormone responsive elements in StDREBs. The ABRE showed the highest frequency followed by ERE, CGTCA-motif and TGACG-motif, respectively.

Phytohormones mediate several crucial biological processes in plant development and abiotic/biotic stress response cascades [87]. Motifs involved in hormonal regulation were found to be the fourth largest including ABRE, AuxRR-core, AuxRE, CGTCA-motif, ERE, GARE-motif, P-box, TATC-box, TCA-element, TGA-box, TGACG-element, and TGA-element, which revealed their association with hormone specific expression. Fig 8 shows the maximum abundance of ABRE in DREB genes while AuxRE was found in the lowest percentage. Our results revealed the role of the abscisic acid (ABA) responsive element (ABRE) motif in dehydration and salinity stress. The motif ABRE was found in 59 out of 66 StDREB gene promoters. It has long been reported that ABA plays a significant role in plant defense against abiotic stress. ABA is known to drive short-term responses such as stomatal closure in response to osmotic circumstances like drought and excessive salinity, resulting in water balance maintenance and long-term growth responses through modulation of stress-responsive genes [88]. Our results demonstrated three auxin-responsive cis-elements i.e. AuxRR-core, AuxRE, and TGA-box. Auxin is a multi-functional hormone given that it is the only one with its long-distance transport system and has an impact on all aspects of plant life including embryogenesis, cell division, plant architecture, and spatial orientation [89]. Auxin performs a vital function during the formation of potato tubers. It has been shown to stimulate tuber initiation and development, and its crosstalk with gibberellic acid and strigolactone in belowground stolon massively increases potato tuber yield [90]. Furthermore, two motifs (CGTCA and TGACG) were found to be methyl-jasmonate (MeJA) responsive whereas three motifs (GARE-motif, P-box, and TATC-box) were responsible for gibberellic acid-responsiveness. Both CGTCA and TGACG were found in 45 StDREB promoters. Methyl jasmonate (MeJA) is an indispensable cellular regulator for a variety of processes including stress tolerance, leaf senescence, and seed germination [91]. Our results also revealed the presence of ethylene-responsive (ERE) and salicylic acid-responsive cis-elements (TCA-element), which existed in 53 and 29 StDREB promoters, respectively. Plants’ anti-oxidative protection mechanism is triggered by ethylene, which leads to a reduction in oxidative stress, accompanied by a recovery in plant growth and photosynthetic efficiency [92]. Salicylic acid (SA) is an anti-oxidant that has important functional roles in abiotic stresses such as drought, salinity, and freezing stress [93]. Of these identified hormone regulatory cis-elements, ABRE and ERE were significantly abundant in 66 StDREB gene promoters. Our findings proposed that StDREBs interact with these hormones and may be directly or indirectly responsible for the induction of various stress-related responses. Similar phytohormone responsive cis-elements were reported in *Saccharum spontaneum* DREB gene promoters while *Ananas comosus* (L.) constituted ABRE only. Detailed promoter frequency has been summarized in S2 Table.

Among 104 CREs, 12.5% were annotated as motifs with unknown functions including MYB_like sequence, Box II-like sequence, Box III, CTAG-motif, CCGTCC motif, TGA, unnamed__2, unnamed__4, unnamed__6, unnamed__8, unnamed_10, unnamed__12, and unnamed__14 (S1 and S2 Tables). Consequently, the presence of various cis-regulatory elements (CREs) in the transcription factor binding sites of StDREB genes showed that the DREB genes had multiple roles in growth, metabolism, and stress-related pathways in Potato.

### Protein modeling, model quality assessment, and validation

The six representative StDREB proteins were selected from the A1-A6 subgroups for three-dimensional protein modeling. The SOPMA online web-server was used to assess secondary structures of the StDREB proteins. The percentage of α-helices, extended strand, beta-turn, and random coil were observed between 21.83–51.64%, 5.74–11.50%, 1.64–4.14%, and 40.98–64.01%, respectively (Table 2). The variations in proteins have a wide range of effects on protein sequence, structure, stability, abundance, and activity. These structural variations could provide StDREB proteins with functional diversity and structural versatility. DREB protein structure analysis showed that in addition to α-helices and Beta turns random coils also form a big chunk of DREB proteins. As in random coils, the confirmation of every amino acid in the backbone chain is independent of the neighboring residues’ confirmation. So, the characteristics of random coils are solely dependent on the intrinsic molecular features of the polypeptide chain. Thus it can be hypothesized that the spatial arrangement of DREB proteins can be provisionally changed based on the protein’s intrinsic nature, regulated by environmental and metabolic variations, attributing to the diverse functional variations of DREB [94]. Such studies have recently been performed in wheat and rice [95, 96].

**Table 2:** Secondary structure analysis of StDREB proteins using SOPMA server.

DREB subgroups	Proteins	α–helices (%)	Extended strand (%)	Beta turn (%)	Random coil (%)
A1	StDREB59	51.64	5.74	1.64	40.98
A2	StDREB15	30.67	7.03	2.88	59.42
A3	StDREB64	24.14	10.69	4.14	61.03
A4	StDREB32	44.32	10.80	2.84	42.05
A5	StDREB12	33.10	11.03	2.76	53.10
A6	StDREB44	21.83	11.50	2.65	64.01

**Fig 9:**
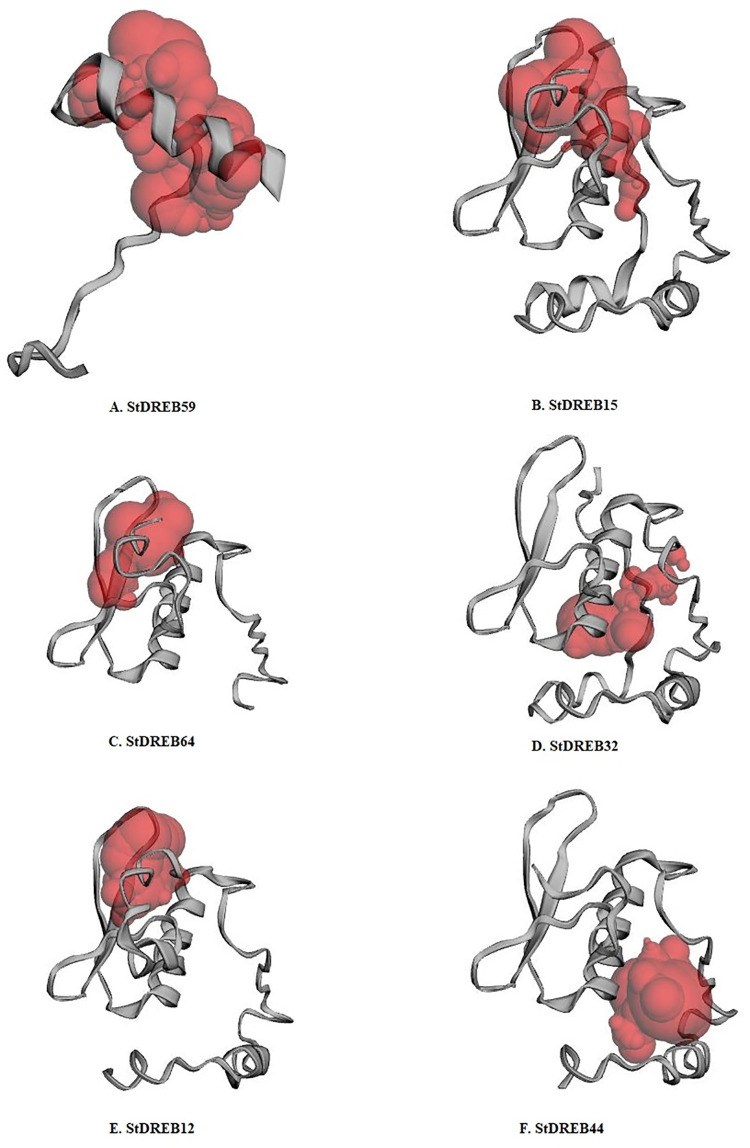
Predicted 3D structures and pocket binding sites of the StDREB proteins. The putative 3D structures of StDREB proteins were generated by the Phyre2 server and refined by the Galaxy Refine server. The active catalytic binding sites were illustrated as red regions using the CASTp server 3.0.

Generating a protein’s 3D structure is important to fill in the gap between protein sequence and protein structure. The generation of 3D models gives insights about protein structure, function, localization, and its interaction network [97]. Phyre2 online server was employed to predict 3D structures of StDREB proteins based on the homology modeling principle. Structural variations were detected in 3D topologies. The models with high confidence and identity percentage were selected (S3 Table). For StDREB59, 34 residues (28% of our sequence) were modeled by the single highest scoring template. A total of 125 residues i.e. 40% of our sequence were modeled for the protein StDREB15. In the case of StDREB64, StDREB32, StDREB12, and StDREB44 about 80 residues (28% our sequence), 113 residues (64% of our sequence), 102 residues (70% of our sequence), and 94 residues (28% of our sequence) were modeled by the best scoring template, respectively. The obtained models were submitted to the Galaxy Refine server for further refinement, which rebuilds all side-chain conformations preceded by overall structure relaxation. The quality of the predicted 3D models was evaluated using the PROCHECK server with Ramachandran Plot analysis where >90% of residues were allowed in the favorable region. Validation of three-dimensional structures has been considered as the core of structural determination methods [98]. The number of residues in favored regions was identified as 92.9% for StDREB59, 90.7% for StDREB15, 92.5% for StDREB64, 94.7% for StDREB32, 95.7% for StDREB12, and 93.6% for StDREB44. Thus, the calculated percentages for Ramachandran Plot verified the good stereo-chemical quality of the generated models (Fig 9).

### Predicted pocket binding sites and disordered regions of StDREB proteins

Molecular pockets were detected on the StDREB protein surfaces using CASTp 3.0 server, which locates, delineates, and measures concave regions on 3D structures of proteins. Protein binding pocket detection is considered vital for structural versatility and interaction specificity. Many structural studies, such as functional site prediction, include the identification of ligand binding sites in protein structures [99]. Therefore, we predicted pocket binding sites of StDREB proteins to gain insights into the structural variations that might be connected with the functional roles of DREB proteins in *S*. *tuberosum*. The active catalytic ligand binding sites were illustrated as red regions (Fig 9). Detailed surface area and volumes were also measured for predicted ligand binding sites (Table 3). The amino acid residues surrounding a protein’s binding pocket determine its physiochemical properties and form, as well as its functionality [100]. Numerous functionally significant residues were localized in this pocket, including valine (VAL), glutamine (GLN), arginine (ARG), asparagine (ASN), glycine (GLY), lysine (LYS), alanine (ALA), aspartic acid (ASP), glutamic acid (GLU), tyrosine (TYR), isoleucine (ILE), methionine (MET), and leucine (LEU).

**Table 3:** Predicted area and volume of the StDREB proteins’ active catalytic site using CASTp server.

DREB subgroups	Protein	Area (Solvent accessible surface-SA)	Volume (Solvent accessible surface-SA)
A1	StDREB59	630.940	374.969
A2	StDREB15	493.984	431.147
A3	StDREB64	297.723	244.975
A4	StDREB32	218.213	97.534
A5	StDREB12	322.427	251.415
A6	StDREB44	308.613	294.684

**Fig 10:**
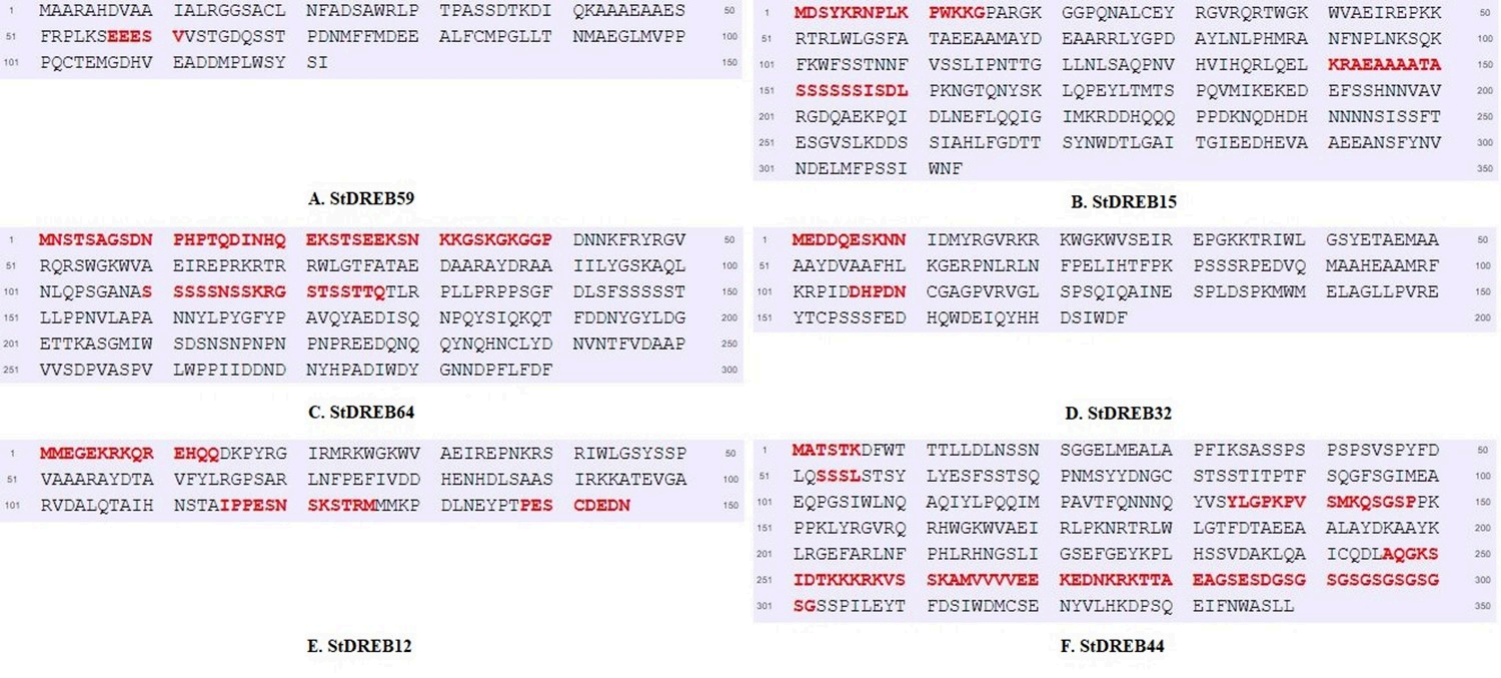
Predicted disordered regions (DRs) of StDREB proteins using the PrDOS server. The red residues represented the disordered regions in the amino acid sequences.

Disordered regions are connected with key cellular processes such as cell cycle control, signaling cascades, transcription regulation, and chaperone activity [101]. In addition, disordered proteins’ intrinsic conformational versatility gives them a strong molecular advantage in transient interactions, allowing them to bind multiple partners with low affinity and high specificity [102]. Disordered regions (DRs) of the StDREB proteins were identified and indicated as red by the PrDOS web program (Fig 10).

### Protein association network of StDREBs

Protein biological functions and molecular pathways could be better understood by interaction network analysis. Protein-protein interaction analysis reveals valuable information about a protein’s unknown function [103]. Therefore, we employed STRING to predict the proteins that interact with StDREBs. The interactions consisted of a full network based on physical and functional protein associations. The findings revealed numerous interactions of six StDREB proteins with regulatory proteins encoded by several other genes not related to the DREB gene family.

**Fig 11:**
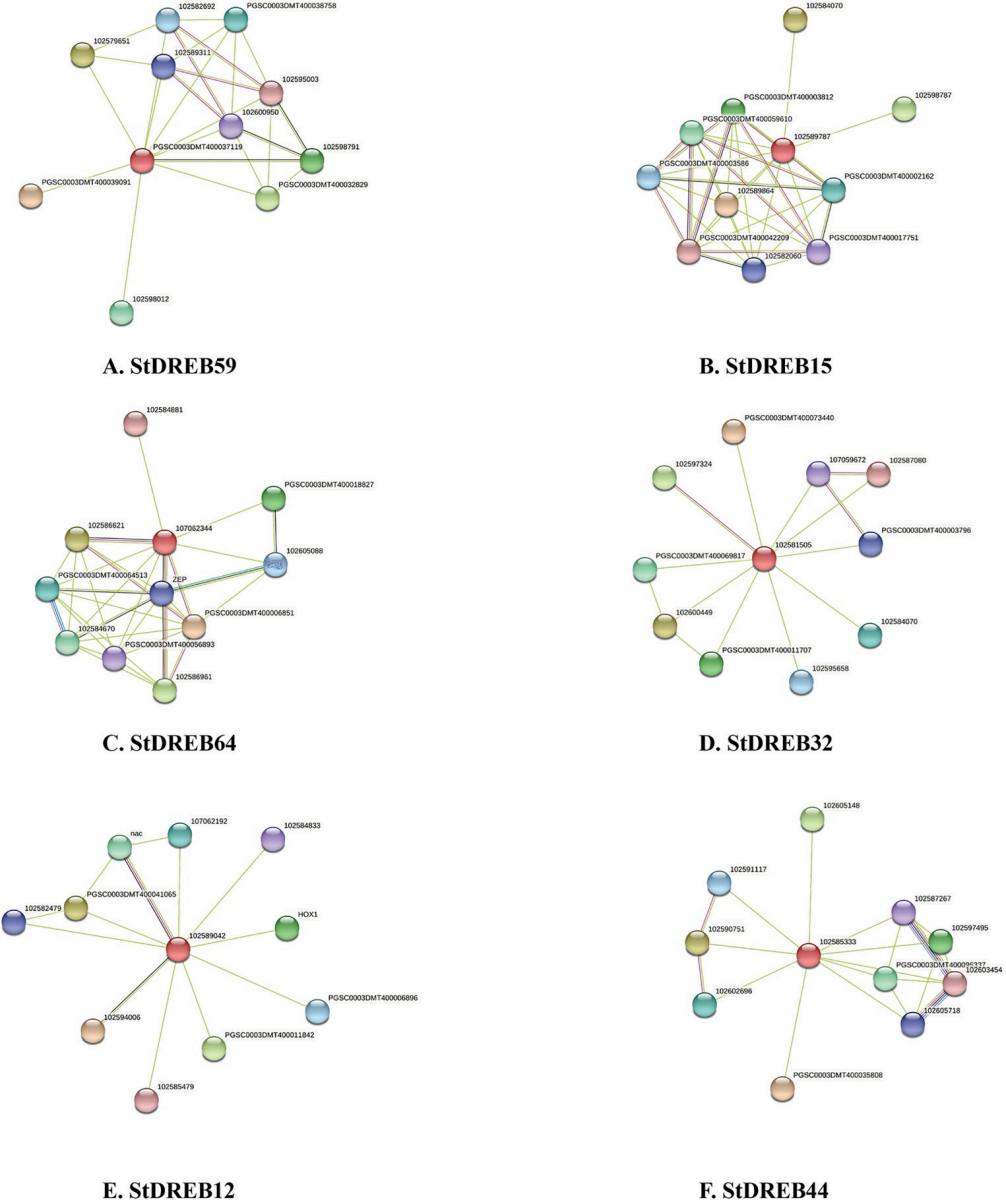
Protein-protein interaction networks of (A) StDREB59, (B) StDREB15, (C) StDREB64, (D) StDREB32, (E) StDREB12, and (F) StDREB44 detected through STRING tool. The red node indicated six query proteins from A1-A6 subgroups and other nodes represented their predicted functional partners. The light green, magenta, black, green, aqua, blue, and light sky blue lines represented protein-protein associations predicted by text-mining, experimental determination, co-expression, gene neighborhood, gene co-occurrence, curated databases, and protein homology respectively.

Our results demonstrated the direct interaction of StDREB59 (PGSC0003DMT400037119) with ten other potential protein partners (Fig 11A). Based on UNIPROT information, two (102598791 and PGSC0003DMT400032829) members were found to be NAC-domain proteins. NAC-domain-containing proteins belong to one of the largest transcription factor families and play essential roles in embryo development, cell cycle regulation, lateral root formation, flowering, and hormone signaling, as well as biotic and abiotic stress responses [104, 105]. The results indicated the interaction of two transmembrane proteins (THM18) (102600950 and 102595003) with StDREB59. According to Pfam results, THM18 proteins are characterized by the presence of two SANT domains, which are considered essential for histone acetyltransferase activity [106]. Histone acetylation is a key phase in the epigenetic regulation of many biological processes, including plant stress responses. Histone acetyltransferases are important regulators of cell differentiation, influencing cell cycle progression, gene interplay, and plant responses to environmental cues [107, 108]. For instance, genome-wide analyses of HATs have been performed in rice [109], tomato [110], brachypodium [111], and cotton [112]. Expression profiling of histone acetyltransferases and histone deacetylases regulated drought stress responses in wheat [113]. Two important bHLH transcription factor family members (102582692 and 102589311) were identified interacting with StDREB59, suggesting their mutual role in signal transduction and stress responses. bHLH TFs play roles in a variety of anabolic mechanisms and signal transduction in plants, including anthocyanin synthesis, gibberellin synthesis, light signaling, and tryptophan biosynthesis [28]. They also regulate stress responses, such as drought, salinity, heat, and cold, thereby regulating the plant’s adaptive responses [114]. Another interacting protein Calmodulin-binding transcription activator (Camta) (PGSC0003DMT400038758) was also recognized interacting with StDREB59 protein. Calmodulins (CaMs) are calcium-binding proteins that bind to a large number of target proteins such as metabolic enzymes, protein kinases, ion channels, protein phosphatases, and transporters [115]. N-carbamyl-L-amino acid amidohydrolase (PGSC0003DMT400039091), a plant enzyme involved in allantoin, purine nucleobase, and ureide catabolic process were also associated with the StDREB59 protein network. Interestingly, StDREB59 also interacted with StDREB9 (PGSC0003DMT400061541, 102579651) which belongs to A2 subgroup, suggesting that *S*. *tuberosum* DREB proteins also interact with each other to regulate various stress responses. An interesting protein MAEWEST (102598012) was also found in the interaction network. The MAEWEST protein belongs to the WUSCHEL-related homeobox (WOX) transcription factor family, which is characterized by the presence of a conserved DNA-binding homeodomain and participates in key developmental processes such as organ formation [116]. The second protein StDREB15 (PGSC0003DMT400060970; 102589787) belongs to the A2 subgroup of the StDREB gene family. Like StDREB59, StDREB15 also interacted with a member of the bHLH transcription factor family (102598787). In addition, LOB domain-containing protein (102584070), Homeobox protein (PGSC0003DMT400017751), Zinc-finger protein (PGSC0003DMT400042209), DNA binding proteins (PGSC0003DMT400002162; 102589864; PGSC0003DMT400003586), protein phosphatase 2c (PGSC0003DMT400059610; PGSC0003DMT400003812), and Brassinazole-resistant 1 protein (102582060) were revealed in protein network, indicating their linked functional associations (Fig 11B). Based on the functional annotation of potential interacting partners, LOB domain-containing protein mediates hormone signaling, Brassinazole-resistant 1 protein facilitates brassinosteroid mediated signaling pathways which has vital role in plant physiology and development, protein phosphatase 2c has important molecular functions in serine/ threonine phosphatase activity and they acts as binding sites for several cofactors, whereas Zinc-finger protein is known to increase abiotic stress tolerance by regulating ROS scavenging, osmotic potential, as well as potassium and sodium homeostasis. Lateral organ boundaries (LOB) domain (LBD) genes are a gene family that encodes plant specific TFs and are important in plant growth and development [117]. LBD genes are vital modulators of plant lateral organ development such as inflorescence, leaf, embryo, root, as well as hormone signaling and stress responsive, implying that these proteins act in a variety of processes [118]. For instance, LBD40 was highly expressed in tomato roots and fruit. SlLBD40 expression was found to be JA dependent, and it may be downstream of MYC2, which is the key TF in JA signaling pathway [119]. Functional characterization of LBD genes in tomatoes identified four genes (SlLBD20, SlLBD47, SlLBD3, and SlLBD22) to be involved in fruit ripening and one gene (SlLBD5) in hormone signaling [120]. Homeobox genes contain a classic DNA binding domain referred to as homeodomain that is required for plant development by controlling cell division and differentiation e.g. WOX [121]. In rice, functional analysis of two stress responsive genes (OsHOX22 and OsHOX24) was found to be highly upregulated under a variety of abiotic stress conditions. Their transcript levels were significantly increased in response to hormones such as SA, Aux, ABA, and GA [122]. Zinc finger proteins are important transcriptional regulators in plant responses to a variety of stress conditions such as drought, salt, heat, light, and oxidative stress [123]. PP2Cs play an important role in ABA signaling and stress responses. RNA sequences and qRT-PCR analysis suggested that potato PPP2C genes respond to multiple abiotic stresses, particularly salinity and water stress [124]. Plant growth and development have been linked to the BZR gene family. Expression profiling in wheat revealed the role of TaBZRs in various developmental stages as well as stress tolerance to cold, drought, and heat [125]. Similarly, various proteins were predicted to associate with A3 subgroup protein i.e. StDREB64 (PGSC0003DMT400085168, 107062344) (Fig 11C). The regulatory proteins included Sodium transporter hkt1 (102584881), Pentatricopeptide repeat-containing protein (PGSC0003DMT400018827), DNA-binding protein (PGSC0003DMT400006851), ABI3 (102586961), short-chain dehydrogenase reductase (PGSC0003DMT400064513; 102584670), VP1-ABI3 (102586621), and Molybdenum cofactor sulfurase (PGSC0003DMT400056893), which are responsible for sodium ion transmembrane transporter activity, biotic and abiotic stress responses, sequence-specific TF binding sites, abscisic acid responsiveness, xanthoxin dehydrogenase activity, seed maturation, and pyridoxal phosphate binding respectively. HKT1 makes a significant contribution to salinity tolerance by removing sodium ion from the transcription stream in *Arabidopsis* [126]. PPR proteins are directed to chloroplast or mitochondria and affect their expression by modifying RNA processing. Their combined action has a significant impact on plant development, respiration, photosynthesis, and environmental responses [127]. Furthermore, Mg protoporphyrin IX chelatase (102605088) has diverse biological and molecular function such as involvement in chlorophyll biosynthetic process and ATP binding, respectively. Mg chelatase is a polymeric enzyme which introduces a magnesium ion into protoporphyrin IX to produce Mg protoporphyrin IX during the first committed step of chlorophyll synthesis [128]. The protein, Zeaxanthin epoxidase (PGSC0003DMT400010287, ZEP) exhibits catalytic activity for the conversion of zeaxanthin into antheraxanthin and subsequently violaxanthin, a key reaction for abscisic acid biosynthesis [129]. The gene ontology biological processes indicated the response of zeaxanthin epoxidase to heat, red light, and water deprivation as well.

The results revealed that the StDREB32 (PGSC0003DMT400046516, 102581505) demonstrated the association with Eukaryotic translation initiation factor 3 subunit (PGSC0003DMT400073440), MYB-like DNA-binding domain protein (102587080), MYB transcription factor (PGSC0003DMT400003796), LOB domain-containing protein (102584070), Stachyose synthase (102595658), and EMB2757 (102597324) (Fig 11D). MYB transcription factors are known for their role in hormone signal transduction and abiotic stress tolerance [130]. The protein EMB2757 exhibits numerous functional roles such as embryo development, root development, desiccation tolerance, and cellular response to DNA damage stimulus and plasmodesmata organization whereas; Stachyose synthase participates in carbohydrate metabolism. Stachyose is a tetra-saccharide that is known as a transport carbohydrate [131]. The raffinose family of oligosaccharides (RFOs) (Ajugose, Verbascose, and Stachyose)protect the embryo from maturation related desiccation, act as a signaling molecule after pathogen attack accrues in vegetative tissues in response to a variety of abiotic stresses [132]. Four proteins were annotated as conserved genes with unknown functions. Based on Pfam’s description, two proteins (107059672; 102600449) possessed the DUF241 domain and DUF761 respectively, which represent plant proteins with unknown functions. The remaining two proteins (PGSC0003DMT400069817, PGSC0003DMT400011707) lacked the presence of domain. The STRING interaction revealed the association of StDREB12 (PGSC0003DMT400020525, 102589042) with NAC domain protein (PGSC0003DMT400082472, nac), Homeobox protein knotted-1-like LET12 (HOX1), Chromatin remodeling complex subunit (PGSC0003DMT400011842), RelA-SpoT RSH4 (102594006), and APETALA2 (102582479) (Fig 11E). RelA-SpoT RSH4 (domain HD_4) protein regulates transcriptional and translational activities, plant development, and stress responses [133]. Two out of ten proteins were identified as Avr9/Cf-9 rapidly elicited protein 65 (102584833; 102585479), which are immediately activated upon the perception of the pathogen. Additionally, StDREB12 also interacted with StDREB13 (PGSC0003DMT400041065), which suggested their involvement in a similar protein network. Based on the results, another protein with bHLH domain (107062192) had a role in protein dimerization activity while the tenth protein (PGSC0003DMT400006896) lacked the Pfam domain and its role remained unclear. Furthermore, protein interactions of StDREB44 (PGSC0003DMT400022023, 102585333) were analyzed. Among ten interacting proteins, three were found to be histone deacetylase (102587267, 102603454, and 102605718). The HD type 1 subfamily of histone deacetylases is involved in plant growth, development, and abiotic and biotic stress responses. Other proteins involved Leucine-rich repeats (102602696; 102591117), magnesium and cobalt efflux protein corC (10259075), Protein LE25 (102605148), Caleosin (PGSC0003DMT400096337; 102597495), and AP2 domain-containing transcription factor (PGSC0003DMT400035808) (Fig 11F). Late embryogenesis abundant (LE25) proteins act as protector molecules under water and salt stress [134] whereas Caleosin protein is responsible for calcium ion binding in signal transduction [135]. Overall, our findings suggested that StDREB proteins directly interact with various proteins encoded by different gene families, and are possibly involved in plant growth, development, and abiotic and biotic stress tolerance. A deep investigation of these proteins will expand the function and interaction network of StDREBs.

## Conclusions

The present study illustrates a detailed in-silico investigation of the StDREB gene promoter regions. We identified and categorized 104 cis-regulatory promoter elements into seven functional groups. Our results elucidated varied motifs which were found to be involved in various functions such as abiotic stress responses (drought and low temperature), hormonal induction, light regulation, cell development, promoter binding sites, and biotic stress responses. The results of homology modeling provided a detailed insight into secondary and tertiary structures of StDREB proteins, as well as catalytic binding sites and disordered regions. Our results confirmed that structural validation of the predicted protein models was reliable and consistent. Moreover, our results indicated protein interactions among StDREBs and their potential interacting partners, which suggested their putative functional diversity. Taken together, our findings shed light on the possible roles of the *S*. *tuberosum* DREB gene family. Our data will serve as a solid foundation for unraveling the candidate genes required for genetic engineering to enhance stress tolerance in *S*. *tuberosum*, as well as a better understanding of the functional relationships between StDREB genes.

## Supporting information

S1 Table Functions of identified cis-regulatory elements in sixty-six (66) StDREB genes. (XLSX)

S2 Table The number of cis-regulatory motifs in sixty-six (66) StDREB gene promoters. (XLSX)

S3 Table Detailed template information for StDREB protein modeling using Phyre2 web server. (XLSX)

## References

[1] Handayani T, Gilani SA, Watanabe KN. Climatic changes and potatoes: How can we cope with the abiotic stresses? Breed Sci. 2019;69(4):545–63. doi: 10.1270/jsbbs.19070 31988619 PMC6977456

[2] Khalil T, Asad SA, Khubaib N, Baig A, Atif S, Umar M, et al. Climate change and potential distribution of potato (Solanum tuberosum) crop cultivation in Pakistan using Maxent. AIMS Agric Food. 2021;6(2):663–76. 10.3934/agrfood.2021039.

[3] Demirel U, Morris WL, Ducreux LJ, Yavuz C, Asim A, Tindas I, et al. Physiological, Biochemical, and Transcriptional Responses to Single and Combined Abiotic Stress in Stress-Tolerant and Stress-Sensitive Potato Genotypes. Front Plant Sci. 2020;11:169. doi: 10.3389/fpls.2020.00169 32184796 PMC7058966

[4] Akhtar M, Jaiswal A, Taj G, Jaiswal J, Qureshi M, Singh N. DREB1/CBF transcription factors: their structure, function and role in abiotic stress tolerance in plants. J Genet. 2012;91(3):385–95. doi: 10.1007/s12041-012-0201-3 23271026

[5] Lahlou O, Ledent J-F. Root mass and depth, stolons and roots formed on stolons in four cultivars of potato under water stress. Eur J Agron. 2005;22(2):159–73. 10.1016/j.eja.2004.02.004.

[6] Aliche EB, Oortwijn M, Theeuwen TP, Bachem CW, Visser RG, van der Linden CG. Drought response in field grown potatoes and the interactions between canopy growth and yield. Agric Water Manag. 2018;206:20–30. 10.1016/j.agwat.2018.04.013.

[7] Schittenhelm S, Sourell H, Löpmeier F-J. Drought resistance of potato cultivars with contrasting canopy architecture. Eur J Agron. 2006;24(3):193–202. 10.1016/j.eja.2005.05.004.

[8] Hijmans RJ. The effect of climate change on global potato production. Am J Pot Res. 2003;80(4):271–9. 10.1007/BF02855363.

[9] Wang Y, Lu W, Deng D. Bioinformatic landscapes for plant transcription factor system research. Planta. 2016;243(2):297–304. https://link.springer.com/article/10.1007/s00425-015-2453-7 26719053 10.1007/s00425-015-2453-7

[10] Lindemose S, O’Shea C, Jensen MK, Skriver K. Structure, function and networks of transcription factors involved in abiotic stress responses. Int J Mol Sci. 2013;14(3):5842–78. doi: 10.3390/ijms14035842 23485989 PMC3634440

[11] Rae L, Lao NT, Kavanagh TA. Regulation of multiple aquaporin genes in Arabidopsis by a pair of recently duplicated DREB transcription factors. Planta. 2011;234(3):429–44. doi: 10.1007/s00425-011-1414-z 21509693

[12] Dong C, Xi Y, Chen X, Cheng Z-M. Genome-wide identification of AP2/EREBP in Fragaria vesca and expression pattern analysis of the FvDREB subfamily under drought stress. BMC Plant Biol. 2021;21(1):1–14. doi: 10.1186/s12870-020-02777-7 34174836 PMC8236174

[13] Dossa K, Wei X, Li D, Fonceka D, Zhang Y, Wang L, et al. Insight into the AP2/ERF transcription factor superfamily in sesame and expression profiling of DREB subfamily under drought stress. BMC Plant Biol. 2016;16(1):1–16. doi: 10.1186/s12870-016-0859-4 27475988 PMC4967514

[14] Zandkarimi H, Ebadi A, Salami SA, Alizade H, Baisakh N. Analyzing the expression profile of AREB/ABF and DREB/CBF genes under drought and salinity stresses in grape (Vitis vinifera L.). PLoS One. 2015;10(7):e0134288. doi: 10.1371/journal.pone.0134288 26230273 PMC4521911

[15] Niu X, Luo T, Zhao H, Su Y, Ji W, Li H. Identification of wheat DREB genes and functional characterization of TaDREB3 in response to abiotic stresses. Gene. 2020;740:144514. doi: 10.1016/j.gene.2020.144514 32112985

[16] Lan Thi Hoang X, Du Nhi NH, Binh Anh Thu N, Phuong Thao N, Phan Tran L-S. Transcription factors and their roles in signal transduction in plants under abiotic stresses. Curr Genomics. 2017;18(6):483–97. doi: 10.2174/1389202918666170227150057 29204078 PMC5684650

[17] Dhatterwal P, Basu S, Mehrotra S, Mehrotra R. Genome wide analysis of W-box element in Arabidopsis thaliana reveals TGAC motif with genes down regulated by heat and salinity. Sci Rep 2019;9(1):1–8. doi: 10.1038/s41598-018-37186-2 30737427 PMC6368537

[18] Hernandez-Garcia CM, Finer JJ. Identification and validation of promoters and cis-acting regulatory elements. Plant Sci. 2014;217:109–19. doi: 10.1016/j.plantsci.2013.12.007 24467902

[19] Ho C-L, Geisler M. Genome-wide computational identification of biologically significant cis-regulatory elements and associated transcription factors from rice. Plants. 2019;8(11):441. doi: 10.3390/plants8110441 31652796 PMC6918188

[20] Sharma N, Russell SD, Bhalla PL, Singh MB. Putative cis-regulatory elements in genes highly expressed in rice sperm cells. BMC Res Notes. 2011;4(1):1–10. doi: 10.1186/1756-0500-4-319 21892935 PMC3224587

[21] Kaur G, Pati PK. Analysis of cis-acting regulatory elements of respiratory burst oxidase homolog (Rboh) gene families in Arabidopsis and rice provides clues for their diverse functions. Comput Biol Chem. 2016;62:104–18. doi: 10.1016/j.compbiolchem.2016.04.002 27111707

[22] Lescot M, Déhais P, Thijs G, Marchal K, Moreau Y, Van de Peer Y, et al. PlantCARE, a database of plant cis-acting regulatory elements and a portal to tools for in silico analysis of promoter sequences. Nucleic Acids Res. 2002;30(1):325–7. doi: 10.1093/nar/30.1.325 11752327 PMC99092

[23] Chow C-N, Zheng H-Q, Wu N-Y, Chien C-H, Huang H-D, Lee T-Y, et al. PlantPAN 2.0: an update of plant promoter analysis navigator for reconstructing transcriptional regulatory networks in plants. Nucleic Acids Res. 2016;44(D1):D1154–D60. doi: 10.1093/nar/gkv1035 26476450 PMC4702776

[24] Shahmuradov IA, Gammerman AJ, Hancock JM, Bramley PM, Solovyev VV. PlantProm: a database of plant promoter sequences. Nucleic Acids Res. 2003;31(1):114–7. doi: 10.1093/nar/gkg041 12519961 PMC165488

[25] Davuluri RV, Sun H, Palaniswamy SK, Matthews N, Molina C, Kurtz M, et al. AGRIS: Arabidopsis gene regulatory information server, an information resource of Arabidopsis cis-regulatory elements and transcription factors. BMC Bioinform. 2003;4(1):1–11. doi: 10.1186/1471-2105-4-25 12820902 PMC166152

[26] Higo K, Ugawa Y, Iwamoto M, Korenaga T. Plant cis-acting regulatory DNA elements (PLACE) database: 1999. Nucleic Acids Res. 1999;27(1):297–300. doi: 10.1093/nar/27.1.297 9847208 PMC148163

[27] Berendzen KW, Weiste C, Wanke D, Kilian J, Harter K, Dröge-Laser W. Bioinformatic cis-element analyses performed in Arabidopsis and rice disclose bZIP-and MYB-related binding sites as potential AuxRE-coupling elements in auxin-mediated transcription. BMC Plant Biol. 2012;12(1):1–19. doi: 10.1186/1471-2229-12-125 22852874 PMC3438128

[28] Zhang Z, Chen J, Liang C, Liu F, Hou X, Zou X. Genome-Wide Identification and Characterization of the bHLH Transcription Factor Family in Pepper (Capsicum annuum L.) Front genet. 2020;11:1156. 10.3389/fgene.2020.570156. PMC7545091 33101390

[29] Huang S-h, Liu Y-x, Deng R, Lei T-t, Tian A-j, Ren H-h, et al. Genome-wide identification and expression analysis of the GSK gene family in Solanum tuberosum L. under abiotic stress and phytohormone treatments and functional characterization of StSK21 involvement in salt stress. Gene. 2021;766:145156. doi: 10.1016/j.gene.2020.145156 32949696

[30] Mirzaei K, Bahramnejad B, Fatemi S. Genome-wide identification and characterization of the bZIP gene family in potato (Solanum tuberosum). Plant Gene. 2020:100257. 10.1016/j.plgene.2020.100257

[31] Zhou Y, Zhou W, Liu H, Liu P, Li Z. Genome‐wide analysis of the soybean DREB gene family: Identification, genomic organization and expression profiles in response to drought stress. Plant Breed. 2020;139(6):1158–67. 10.1111/pbr.12867.

[32] Liu X, Zhu J, Wei C, Guo Q, Bian C, Xiang Z, et al. Genome-wide identification and characterization of the DREB transcription factor gene family in mulberry. Biol Plant. 2015;59(2):253–65. 10.1007/s10535-015-0498-x.

[33] Zhao T, Liang D, Wang P, Liu J, Ma F. Genome-wide analysis and expression profiling of the DREB transcription factor gene family in Malus under abiotic stress. Mol Genet Genom 2012;287(5):423–36. doi: 10.1007/s00438-012-0687-7 22526429

[34] Konzen ER, Recchia GH, Cassieri F, Caldas DGG, Berny Mier y Teran JC, Gepts P, et al. DREB genes from common bean (Phaseolus vulgaris L.) show broad to specific abiotic stress responses and distinct levels of nucleotide diversity. Int J Genomics. 2019;2019. doi: 10.1155/2019/9520642 31249842 PMC6525893

[35] Bouaziz D, Pirrello J, Charfeddine M, Hammami A, Jbir R, Dhieb A, et al. Overexpression of StDREB1 transcription factor increases tolerance to salt in transgenic potato plants. Mol Biotechnol. 2013;54(3):803–17. doi: 10.1007/s12033-012-9628-2 23250722

[36] Li W, Chen Y, Ye M, Lu H, Wang D, Chen Q. Evolutionary history of the C-repeat binding factor/dehydration-responsive element-binding 1 (CBF/DREB1) protein family in 43 plant species and characterization of CBF/DREB1 proteins in Solanum tuberosum. BMC Evol Biol. 2020;20(1):1–14. doi: 10.1186/s12862-019-1549-2 33143637 PMC7607821

[37] Song Q, Wang X, Li J, Chen TH, Liu Y, Yang X. CBF1 and CBF4 in Solanum tuberosum L. differ in their effect on low-temperature tolerance and development. Environ Exp bot. 2021;185:104416. 10.1016/j.envexpbot.2021.104416.

[38] Mushtaq N, Munir F, Gul A, Amir R, Paracha RZ. Genome-wide analysis, identification, evolution and genomic organization of dehydration responsive element-binding (DREB) gene family in Solanum tuberosum. Peer J. 2021;9:e11647. doi: 10.7717/peerj.11647 34221730 PMC8236231

[39] Goodstein DM, Shu S, Howson R, Neupane R, Hayes RD, Fazo J, et al. Phytozome: a comparative platform for green plant genomics. 2012;40(D1):D1178–D86. doi: 10.1093/nar/gkr944 22110026 PMC3245001

[40] Lescot M, Déhais P, Thijs G, Marchal K, Moreau Y, Van de Peer Y, et al. PlantCARE, a database of plant cis-acting regulatory elements and a portal to tools for in silico analysis of promoter sequences. 2002;30(1):325–7. doi: 10.1093/nar/30.1.325 11752327 PMC99092

[41] Consortium U. UniProt: a worldwide hub of protein knowledge. 2019;47(D1):D506–D15. doi: 10.1093/nar/gky1049 30395287 PMC6323992

[42] Geourjon C, Deleage GJB. SOPMA: significant improvements in protein secondary structure prediction by consensus prediction from multiple alignments. 1995;11(6):681–4. doi: 10.1093/bioinformatics/11.6.681 8808585

[43] Kelley LA, Mezulis S, Yates CM, Wass MN, Sternberg MJJNp. The Phyre2 web portal for protein modeling, prediction and analysis. 2015;10(6):845–58. doi: 10.1038/nprot.2015.053 25950237 PMC5298202

[44] Heo L, Park H, Seok CJNar. GalaxyRefine: Protein structure refinement driven by side-chain repacking. 2013;41(W1):W384–W8. doi: 10.1093/nar/gkt458 23737448 PMC3692086

[45] Tian W, Chen C, Lei X, Zhao J, Liang JJNar. CASTp 3.0: computed atlas of surface topography of proteins. 2018;46(W1):W363–W7. doi: 10.1093/nar/gky473 29860391 PMC6031066

[46] Ishida T, Kinoshita KJNar. PrDOS: prediction of disordered protein regions from amino acid sequence. 2007;35(suppl_2):W460–W4. doi: 10.1093/nar/gkm363 17567614 PMC1933209

[47] Szklarczyk D, Gable AL, Lyon D, Junge A, Wyder S, Huerta-Cepas J, et al. STRING v11: protein–protein association networks with increased coverage, supporting functional discovery in genome-wide experimental datasets. Nucleic Acids Res. 2019;47(D1):D607–D13. doi: 10.1093/nar/gky1131 30476243 PMC6323986

[48] Li X, Liang Y, Gao B, Mijiti M, Bozorov TA, Yang H, et al. ScDREB10, an A-5c type of DREB gene of the desert moss Syntrichia caninervis, confers osmotic and salt tolerances to Arabidopsis. Genes. 2019;10(2):146. doi: 10.3390/genes10020146 30769913 PMC6409532

[49] Wang X, Chen X, Liu Y, Gao H, Wang Z, Sun G. CkDREB gene in Caragana korshinskii is involved in the regulation of stress response to multiple abiotic stresses as an AP2/EREBP transcription factor. Mol Biol Rep. 2011;38(4):2801–11. doi: 10.1007/s11033-010-0425-3 21127996

[50] Alves MS, Dadalto SP, Gonçalves AB, De Souza GB, Barros VA, Fietto LG. Plant bZIP transcription factors responsive to pathogens: a review. Int J Mol Sci. 2013;14(4):7815–28. doi: 10.3390/ijms14047815 23574941 PMC3645718

[51] Wang P, Du Y, Zhao X, Miao Y, Song C-P. The MPK6-ERF6-ROS-responsive cis-acting Element7/GCC box complex modulates oxidative gene transcription and the oxidative response in Arabidopsis. Plant Physiol. 2013;161(3):1392–408. doi: 10.1104/pp.112.210724 23300166 PMC3585604

[52] Ganie SA, Ahammed GJ, Wani SH. Vascular plant one zinc-finger (VOZ) transcription factors: novel regulators of abiotic stress tolerance in rice (Oryza sativa L.). Genet Resour Crop Evol. 2020:1–9. 10.1007/s10722-020-00904-9.

[53] Banerjee J, Sahoo DK, Raha S, Sarkar S, Dey N, Maiti IB. A region containing an as-1 element of Dahlia Mosaic Virus (DaMV) subgenomic transcript promoter plays a key role in green tissue-and root-specific expression in plants. Plant Mol Biol Rep. 2015;33(3):532–56. 10.1007/s11105-014-0766-5.

[54] Rinerson CI, Scully ED, Palmer NA, Donze-Reiner T, Rabara RC, Tripathi P, et al. The WRKY transcription factor family and senescence in switchgrass. BMC Genom. 2015;16(1):1–17. doi: 10.1186/s12864-015-2057-4 26552372 PMC4640240

[55] Bae S-H, Han HW, Moon J. Functional analysis of the molecular interactions of TATA box-containing genes and essential genes. PloS One. 2015;10(3):e0120848. doi: 10.1371/journal.pone.0120848 25789484 PMC4366266

[56] Dikstein R. The unexpected traits associated with core promoter elements. Transcription. 2011;2(5):201–6. doi: 10.4161/trns.2.5.17271 22231114 PMC3265775

[57] Porto MS, Pinheiro MPN, Batista VGL, dos Santos RC, de Albuquerque Melo Filho P, de Lima LM. Plant promoters: an approach of structure and function. Mol Biotechnol. 2014;56(1):38–49. doi: 10.1007/s12033-013-9713-1 24122284

[58] Zhao C, Geng X, Yang Y, Chai Y, Song Z, Xi C, et al. NtAIDP1, a novel NtJAZ interacting protein, binds to an AT-rich region to activate the transcription of jasmonate-inducible genes in tobacco. J Plant Physiol. 2021:153452. 10.1016/j.jplph.2021.153452. 34098414

[59] Zhou L, Liu Y, Liu Z, Kong D, Duan M, Luo L. Genome-wide identification and analysis of drought-responsive microRNAs in Oryza sativa. J Exp Bot. 2010;61(15):4157–68. doi: 10.1093/jxb/erq237 20729483

[60] Shariatipour N, Heidari B. Meta-Analysis of Expression of the Stress Tolerance Associated Genes and Uncover their-Regulatory Elements in Rice (L.). Open Bioinform J. 2020;13(1). 10.2174/1875036202013010039.

[61] Gilmartin PM, Sarokin L, Memelink J, Chua N-H. Molecular light switches for plant genes. Plant Cell. 1990;2(5):369. doi: 10.1105/tpc.2.5.369 2152164 PMC159894

[62] Lam E, Chua N-H. ASF-2: a factor that binds to the cauliflower mosaic virus 35S promoter and a conserved GATA motif in Cab promoters. Plant Cell. 1989;1(12):1147–56. doi: 10.1105/tpc.1.12.1147 2535536 PMC159850

[63] Yin G, Xu H, Xiao S, Qin Y, Li Y, Yan Y, et al. The large soybean (Glycine max) WRKY TF family expanded by segmental duplication events and subsequent divergent selection among subgroups. BMC Plant Biol. 2013;13(1):1–19. doi: 10.1186/1471-2229-13-148 24088323 PMC3850935

[64] SAIDI A, HAJIBARAT Z. In silico analysis of floral MADS-BOX gene in Brachypodium distachyon. Bionature. 2018:366–75.

[65] Ma S, Shah S, Bohnert HJ, Snyder M, Dinesh-Kumar SP. Incorporating motif analysis into gene co-expression networks reveals novel modular expression pattern and new signaling pathways. PLoS Genet. 2013;9(10):e1003840. doi: 10.1371/journal.pgen.1003840 24098147 PMC3789834

[66] Liu L, Xu W, Hu X, Liu H, Lin Y. W-box and G-box elements play important roles in early senescence of rice flag leaf. Sci Rep. 2016;6(1):1–9. doi: 10.1038/s41598-016-0001-8 26864250 PMC4749992

[67] Kurt F, Filiz E. Genome-wide and comparative analysis of bHLH38, bHLH39, bHLH100 and bHLH101 genes in Arabidopsis, tomato, rice, soybean and maize: insights into iron (Fe) homeostasis. Biometals. 2018;31(4):489–504. doi: 10.1007/s10534-018-0095-5 29546482

[68] Tanabe N, Tamoi M, Shigeoka S. The sweet potato RbcS gene (IbRbcS1) promoter confers high-level and green tissue-specific expression of the GUS reporter gene in transgenic Arabidopsis. Gene. 2015;567(2):244–50. doi: 10.1016/j.gene.2015.05.006 25958348

[69] Zhao L, Li M, Xu C, Yang X, Li D, Zhao X, et al. Natural variation in Gm GBP 1 promoter affects photoperiod control of flowering time and maturity in soybean. Plant J. 2018;96(1):147–62. doi: 10.1111/tpj.14025 30004144

[70] Kaplan-Levy RN, Brewer PB, Quon T, Smyth DR. The trihelix family of transcription factors–light, stress and development. Trends Plant Sci. 2012;17(3):163–71. doi: 10.1016/j.tplants.2011.12.002 22236699

[71] Wang C, Wang Y, Pan Q, Chen S, Feng C, Hai J, et al. Comparison of Trihelix transcription factors between wheat and Brachypodium distachyon at genome-wide. BMC Genom. 2019;20(1):1–14. doi: 10.1186/s12864-019-5494-7 30770726 PMC6377786

[72] Li N, Wei S, Chen J, Yang F, Kong L, Chen C, et al. Os ASR 2 regulates the expression of a defence‐related gene, Os2H16, by targeting the GT‐1 cis‐element. Plant Biotechnol J. 2018;16(3):771–83. doi: 10.1111/pbi.12827 28869785 PMC5814579

[73] Li R, Zhu F, Duan D. Function analysis and stress-mediated cis-element identification in the promoter region of VqMYB15. Plant Signal Behav 2020;15(7):1773664. doi: 10.1080/15592324.2020.1773664 32475217 PMC8570707

[74] Laloum T, De Mita S, Gamas P, Baudin M, Niebel A. CCAAT-box binding transcription factors in plants: Y so many? Trends Plant Sci. 2013;18(3):157–66. doi: 10.1016/j.tplants.2012.07.004 22939172

[75] Li L, Shi Q, Li Z, Gao J. Genome-wide identification and functional characterization of the PheE2F/DP gene family in Moso bamboo. BMC Plant Biol. 2021;21(1):1–15. doi: 10.1186/s12870-020-02777-7 33781213 PMC8008544

[76] Vulavala VK, Fogelman E, Rozental L, Faigenboim A, Tanami Z, Shoseyov O, et al. Identification of genes related to skin development in potato. Plant Mol Biol. 2017;94(4):481–94. doi: 10.1007/s11103-017-0619-3 28536883

[77] Huang X, Song X, Chen R, Zhang B, Li C, Liang Y, et al. Genome-wide analysis of the DREB subfamily in Saccharum spontaneum reveals their functional divergence during cold and drought stresses. Front genet. 2020;10:1326. doi: 10.3389/fgene.2019.01326 32117408 PMC7013043

[78] Chaudhary R, Baranwal VK, Kumar R, Sircar D, Chauhan H. Genome-wide identification and expression analysis of Hsp70, Hsp90, and Hsp100 heat shock protein genes in barley under stress conditions and reproductive development. Funct Integr Genomics. 2019;19(6):1007–22. doi: 10.1007/s10142-019-00695-y 31359217

[79] Brown RL, Kazan K, McGrath KC, Maclean DJ, Manners JM. A role for the GCC-box in jasmonate-mediated activation of the PDF1. 2 gene of Arabidopsis. Plant Physiol. 2003;132(2):1020–32. doi: 10.1104/pp.102.017814 12805630 PMC167040

[80] Dolferus R, Klok EJ, Ismond K, Delessert C, Wilson S, Good A, et al. Molecular basis of the anaerobic response in plants. IUBMB life. 2001;51(2):79–82. doi: 10.1080/15216540120263 11463167

[81] Li C, Ng CK-Y, Fan L-M. MYB transcription factors, active players in abiotic stress signaling. Environ Exp bot. 2015;114:80–91. 10.1016/j.envexpbot.2014.06.014.

[82] Kaur A, Pati PK, Pati AM, Nagpal AKJPo. In-silico analysis of cis-acting regulatory elements of pathogenesis-related proteins of Arabidopsis thaliana and Oryza sativa. 2017;12(9):e0184523. doi: 10.1371/journal.pone.0184523 28910327 PMC5598985

[83] Yanfang Y, Kaikai Z, Liying Y, Xing L, Ying W, Hongwei L, et al. Identification and characterization of MYC transcription factors in Taxus sp. Gene. 2018;675:1–8. doi: 10.1016/j.gene.2018.06.065 29935357

[84] Niu L, Chu HD, Tran CD, Nguyen KH, Pham HX, Le DT, et al. The GATA gene family in chickpea: structure analysis and transcriptional responses to abscisic acid and dehydration treatments revealed potential genes involved in drought adaptation. J Plant Growth Regul. 2020;39(4):1647–60. 10.1007/s00344-020-10201-5.

[85] Lu Y, Sun J, Yang Z, Zhao C, Zhu M, Ma D, et al. Genome-wide identification and expression analysis of glycine-rich RNA-binding protein family in sweet potato wild relative Ipomoea trifida. Gene. 2019;686:177–86. doi: 10.1016/j.gene.2018.11.044 30453066

[86] Chai M, Cheng H, Yan M, Priyadarshani S, Zhang M, He Q, et al. Identification and expression analysis of the DREB transcription factor family in pineapple (Ananas comosus (L.) Merr.). Peer J. 2020;8:e9006. doi: 10.7717/peerj.9006 32377449 PMC7194095

[87] Shu K, Zhou W, Chen F, Luo X, Yang W. Abscisic acid and gibberellins antagonistically mediate plant development and abiotic stress responses. Front Plant Sci. 2018;9:416. doi: 10.3389/fpls.2018.00416 29636768 PMC5881240

[88] Verma V, Ravindran P, Kumar PP. Plant hormone-mediated regulation of stress responses. BMC Plant Biol. 2016;16(1):1–10. doi: 10.1186/s12870-016-0771-y 27079791 PMC4831116

[89] Kolachevskaya OO, Lomin SN, Arkhipov DV, Romanov GA. Auxins in potato: molecular aspects and emerging roles in tuber formation and stress resistance. Plant Cell Rep. 2019;38(6):681–98. doi: 10.1007/s00299-019-02395-0 30739137

[90] Kondhare KR, Patil AB, Giri AP. Auxin: An emerging regulator of tuber and storage root development. Plant Sci. 2021:110854. doi: 10.1016/j.plantsci.2021.110854 33775360

[91] Concha CM, Figueroa NE, Poblete LA, Oñate FA, Schwab W, Figueroa CR. Methyl jasmonate treatment induces changes in fruit ripening by modifying the expression of several ripening genes in Fragaria chiloensis fruit. Plant Physiol Biochem. 2013;70:433–44. doi: 10.1016/j.plaphy.2013.06.008 23835361

[92] Sharma A, Kumar V, Sidhu GPS, Kumar R, Kohli SK, Yadav P, et al. Abiotic stress management in plants: role of ethylene. 2019:185–208.

[93] Khan MIR, Fatma M, Per TS, Anjum NA, Khan NA. Salicylic acid-induced abiotic stress tolerance and underlying mechanisms in plants. Front Plant Sci. 2015;6:462. doi: 10.3389/fpls.2015.00462 26175738 PMC4485163

[94] Vatansever R, Uras ME, Sen U, Ozyigit II, Filiz E. Isolation of a transcription factor DREB1A gene from Phaseolus vulgaris and computational insights into its characterization: protein modeling, docking and mutagenesis. J Biomol Struct Dyn. 2017;35(14):3107–18. doi: 10.1080/07391102.2016.1243487 27687894

[95] Kumar A, Kumar S, Kumar U, Suravajhala P, Gajula MP. Functional and structural insights into novel DREB1A transcription factors in common wheat (Triticum aestivum L.): A molecular modeling approach. Comput Biol Chem. 2016;64:217–26. doi: 10.1016/j.compbiolchem.2016.07.008 27471160

[96] Nawaz M, Iqbal N, Idrees S, Ullah I. DREB1A from Oryza sativa var. IR6: homology modelling and molecular docking. Turk J Botany 2014;38(6):1095–102.

[97] Contreras-Moreira B, Fitzjohn PW, Bates PA. Comparative modelling: an essential methodology for protein structure prediction in the post-genomic era. Appl Bioinformatics. 2002;1(4):177–90. 15130836

[98] Pražnikar J, Tomić M, Turk D. Validation and quality assessment of macromolecular structures using complex network analysis. Sci Rep. 2019;9(1):1–11. doi: 10.1038/s41598-018-37186-2 30737447 PMC6368557

[99] Kawabata T. Detection of multiscale pockets on protein surfaces using mathematical morphology. Proteins: Struct Funct Genet. 2010;78(5):1195–211. 10.1002/prot.22639. 19938154

[100] Coleman RG, Sharp KA. Protein pockets: inventory, shape, and comparison. J Chem Inf Model. 2010;50(4):589–603. doi: 10.1021/ci900397t 20205445 PMC2859996

[101] Uversky VN. Functional roles of transiently and intrinsically disordered regions within proteins. FEBS J. 2015;282(7):1182–9. doi: 10.1111/febs.13202 25631540

[102] Pazos F, Pietrosemoli N, García-Martín JA, Solano R. Protein intrinsic disorder in plants. Front Plant Sci. 2013;4:363. doi: 10.3389/fpls.2013.00363 24062761 PMC3770944

[103] Braun P, Aubourg S, Van Leene J, De Jaeger G, Lurin C. Plant protein interactomes. Annu Rev Plant Biol. 2013;64:161–87. doi: 10.1146/annurev-arplant-050312-120140 23330791

[104] Singh AK, Sharma V, Pal AK, Acharya V, Ahuja PS. Genome-wide organization and expression profiling of the NAC transcription factor family in potato (Solanum tuberosum L.). DNA Res. 2013;20(4):403–23. doi: 10.1093/dnares/dst019 23649897 PMC3738166

[105] Zhang L, Yao L, Zhang N, Yang J, Zhu X, Tang X, et al. Lateral root development in potato is mediated by stu-mi164 regulation of NAC transcription factor. Front Plant Sci. 2018;9:383. doi: 10.3389/fpls.2018.00383 29651294 PMC5884874

[106] Gao Y, Yang S, Yuan L, Cui Y, Wu K. Comparative analysis of SWIRM domain-containing proteins in plants. Comp Funct Genom. 2012;2012. doi: 10.1155/2012/310402 22924025 PMC3424641

[107] Boycheva I, Vassileva V, Iantcheva A. Histone acetyltransferases in plant development and plasticity. Curr Genomics. 2014;15(1):28. doi: 10.2174/138920291501140306112742 24653661 PMC3958957

[108] Ueda M, Matsui A, Tanaka M, Nakamura T, Abe T, Sako K, et al. The distinct roles of class I and II RPD3-like histone deacetylases in salinity stress response. Plant Physiol. 2017;175(4):1760–73. doi: 10.1104/pp.17.01332 29018096 PMC5717743

[109] Liu X, Luo M, Zhang W, Zhao J, Zhang J, Wu K, et al. Histone acetyltransferases in rice (Oryza sativa L.): phylogenetic analysis, subcellular localization and expression. BMC Plant Biol. 2012;12(1):1–17. doi: 10.1186/1471-2229-12-145 22894565 PMC3502346

[110] Cigliano RA, Sanseverino W, Cremona G, Ercolano MR, Conicella C, Consiglio FM. Genome-wide analysis of histone modifiers in tomato: gaining an insight into their developmental roles. BMC Genom. 2013;14(1):1–20. 10.1186/1471-2164-14-57. PMC3567966 23356725

[111] Tan S, Gao L, Li T, Chen L. Phylogenetic and expression analysis of histone acetyltransferases in Brachypodium distachyon. Genomics. 2019;111(6):1966–76. doi: 10.1016/j.ygeno.2019.01.008 30641128

[112] Imran M, Shafiq S, Farooq MA, Naeem MK, Widemann E, Bakhsh A, et al. Comparative genome-wide analysis and expression profiling of histone acetyltransferase (HAT) gene family in response to hormonal applications, metal and abiotic stresses in cotton. Int J Mol Sci. 2019;20(21):5311. 10.3390/ijms20215311. PMC6862461 31731441

[113] Li H, Liu H, Pei X, Chen H, Li X, Wang J, et al. Comparative Genome-Wide Analysis and Expression Profiling of Histone Acetyltransferases and Histone Deacetylases Involved in the Response to Drought in Wheat. J Plant Growth Regul. 2021:1–14. doi: 10.1007/s00344-021-10335-0 33649694 PMC7905201

[114] Qian Y, Zhang T, Yu Y, Gou L, Yang J, Xu J, et al. Regulatory Mechanisms of bHLH Transcription Factors in Plant Adaptive Responses to Various Abiotic Stresses. Front Plant Sci. 2021;12:1143. doi: 10.3389/fpls.2021.677611 34220896 PMC8250158

[115] Ali E, Raza MA, Cai M, Hussain N, Shahzad AN, Hussain M, et al. Calmodulin-binding transcription activator (CAMTA) genes family: Genome-wide survey and phylogenetic analysis in flax (Linum usitatissimum). PLoS One. 2020;15(7):e0236454. doi: 10.1371/journal.pone.0236454 32702710 PMC7377914

[116] van der Graaff E, Laux T, Rensing SA. The WUS homeobox-containing (WOX) protein family. Genome Biol 2009;10(12):1–9. doi: 10.1186/gb-2009-10-12-248 20067590 PMC2812940

[117] Zhang Y, Li Z, Ma B, Hou Q, Wan X. Phylogeny and functions of LOB domain proteins in plants. Int J Mol Sci. 2020;21(7):2278. doi: 10.3390/ijms21072278 32224847 PMC7178066

[118] Yang H, Shi G, Du H, Wang H, Zhang Z, Hu D, et al. Genome‐Wide Analysis of Soybean LATERAL ORGAN BOUNDARIES Domain‐Containing Genes: A Functional Investigation of GmLBD12. Plant Genome. 2017;10(1):plantgenome2016.07.0058. doi: 10.3835/plantgenome2016.07.0058 28464070

[119] Liu L, Zhang J, Xu J, Li Y, Guo L, Wang Z, et al. CRISPR/Cas9 targeted mutagenesis of SlLBD40, a lateral organ boundaries domain transcription factor, enhances drought tolerance in tomato. Plant Sci. 2020;301:110683. doi: 10.1016/j.plantsci.2020.110683 33218644

[120] Gupta K, Gupta S. Molecular and in silico characterization of tomato LBD transcription factors reveals their role in fruit development and stress responses. Plant Gene. 2021:100309. 10.1016/j.plgene.2021.100309.

[121] Lian G, Ding Z, Wang Q, Zhang D, Xu J. Origins and evolution of WUSCHEL-related homeobox protein family in plant kingdom. Sci World J. 2014;2014. doi: 10.1155/2014/534140 24511289 PMC3913392

[122] Bhattacharjee A, Khurana JP, Jain M. Characterization of rice homeobox genes, OsHOX22 and OsHOX24, and over-expression of OsHOX24 in transgenic Arabidopsis suggest their role in abiotic stress response. Front Plant Sci. 2016;7:627. doi: 10.3389/fpls.2016.00627 27242831 PMC4862318

[123] Wang K, Ding Y, Cai C, Chen Z, Zhu C. The role of C2H2 zinc finger proteins in plant responses to abiotic stresses. Physiol Plant. 2019;165(4):690–700. doi: 10.1111/ppl.12728 29572849

[124] WANG Y-f, LIAO Y-q, WANG Y-p, YANG J-w, ZHANG N, SI H-j. Genome-wide identification and expression analysis of StPP2C gene family in response to multiple stresses in potato (Solanum tuberosum L.). J Integr Agric 2020;19(6):1609–24. 10.1016/S2095-3119(20)63181-1.

[125] Kesawat MS, Kherawat BS, Singh A, Dey P, Kabi M, Debnath D, et al. Genome-wide identification and characterization of the brassinazole-resistant (BZR) gene family and its expression in the various developmental stage and stress conditions in wheat (Triticum aestivum L.). Int J Mol Sci. 2021;22(16):8743. doi: 10.3390/ijms22168743 34445448 PMC8395832

[126] Ali A, Raddatz N, Aman R, Kim S, Park HC, Jan M, et al. A single amino-acid substitution in the sodium transporter HKT1 associated with plant salt tolerance. Plant Physiol. 2016;171(3):2112–26. doi: 10.1104/pp.16.00569 27208305 PMC4936583

[127] Barkan A, Small I. Pentatricopeptide repeat proteins in plants. Annu Rev Plant Biol. 2014;65:415–42. doi: 10.1146/annurev-arplant-050213-040159 24471833

[128] Zhang D, Chang E, Yu X, Chen Y, Yang Q, Cao Y, et al. Molecular characterization of Magnesium Chelatase in soybean [Glycine max (L.) Merr.]. Front Plant Sci. 2018;9:720. doi: 10.3389/fpls.2018.00720 29971071 PMC6018531

[129] Schwarz N, Armbruster U, Iven T, Brückle L, Melzer M, Feussner I, et al. Tissue-specific accumulation and regulation of zeaxanthin epoxidase in Arabidopsis reflect the multiple functions of the enzyme in plastids. Plant Cell Physiol. 2015;56(2):346–57. doi: 10.1093/pcp/pcu167 25416291

[130] Zhang T, Zhao Y, Wang Y, Liu Z, Gao C. Comprehensive analysis of MYB gene family and their expressions under abiotic stresses and hormone treatments in Tamarix hispida. Front Plant Sci. 2018;9:1303. doi: 10.3389/fpls.2018.01303 30283465 PMC6156436

[131] Qiu D, Vuong T, Valliyodan B, Shi H, Guo B, Shannon JG, et al. Identification and characterization of a stachyose synthase gene controlling reduced stachyose content in soybean. Theor Appl Genet. 2015;128(11):2167–76. doi: 10.1007/s00122-015-2575-0 26179337 PMC4624830

[132] Sengupta S, Mukherjee S, Basak P, Majumder AL. Significance of galactinol and raffinose family oligosaccharide synthesis in plants. Front Plant Sci. 2015;6:656. doi: 10.3389/fpls.2015.00656 26379684 PMC4549555

[133] Boniecka J, Prusińska J, Dąbrowska GB, Goc A. Within and beyond the stringent response-RSH and (p) ppGpp in plants. Planta. 2017;246(5):817–42. doi: 10.1007/s00425-017-2780-y 28948393 PMC5633626

[134] Bhardwaj R, Sharma I, Kanwar M, Sharma R, Handa N, Kaur H, et al. LEA proteins in salt stress tolerance. Salt stress in plants: Springer; 2013. p. 79–112.

[135] Song W, Qin Y, Zhu Y, Yin G, Wu N, Li Y, et al. Delineation of plant caleosin residues critical for functional divergence, positive selection and coevolution. BMC Evol Biol. 2014;14(1):1–14. doi: 10.1186/1471-2148-14-124 24913827 PMC4057654

